# Resistant Potato Starch Alters the Cecal Microbiome and Gene Expression in Mice Fed a Western Diet Based on NHANES Data

**DOI:** 10.3389/fnut.2022.782667

**Published:** 2022-03-22

**Authors:** Allen D. Smith, Celine Chen, Lumei Cheung, Robert Ward, Korry J. Hintze, Harry D. Dawson

**Affiliations:** ^1^Diet, Genomics, and Immunology Laboratory, Beltsville Human Nutrition Research Center, Agricultural Research Service, United States Department of Agriculture, Beltsville, MD, United States; ^2^Department of Nutrition, Dietetics and Food Sciences, Utah State University, Logan, UT, United States

**Keywords:** resistant potato starch, microbiota (16S), cecum, colon, gene expression

## Abstract

Several studies indicate that the four major types of resistant starch (RS1-4) are fermented in the cecum and colon to produce short-chain fatty acids (SCFAs) and can alter the microbiome and host physiology. However, nearly all these studies were conducted in rodents fed with a diet that does not approximate what is typically consumed by humans. To address this, mice were fed a Total Western Diet (TWD) based on National Health and Nutrition Examination Survey (NHANES) data that mimics the macro and micronutrient composition of a typical American diet for 6 weeks and then supplemented with 0, 2, 5, or 10% of the RS2, resistant potato starch (RPS), for an additional 3 weeks. The cecal microbiome was analyzed by 16S sequencing. The alpha-diversity of the microbiome decreased with increasing consumption of RPS while a beta-diversity plot showed four discreet groupings based on the RPS level in the diet. The relative abundance of various genera was altered by feeding increasing levels of RPS. In particular, the genus *Lachnospiraceae NK4A136 group* was markedly increased. Cecal, proximal, and distal colon tissue mRNA abundance was analyzed by RNASeq. The cecal mRNA abundance principal component analysis showed clear segregation of the four dietary groups whose separation decreased in the proximal and distal colon. Differential expression of the genes was highest in the cecum, but substantially decreased in the proximal colon (PC) and distal colon (DC). Most differentially expressed genes were unique to each tissue with little overlap in between. The pattern of the observed gene expression suggests that RPS, likely through metabolic changes secondary to differences in microbial composition, appears to prime the host to respond to a range of pathogens, including viruses, bacteria, and parasites. In summary, consumption of dietary RPS led to significant changes to the microbiome and gene expression in the cecum and to a lesser extent in the proximal and distal colon.

## Introduction

Dietary fiber encompasses a broad variety of compounds including polysaccharides, oligosaccharides, lignin, and associated plant substances. Most plant-based foods contain multiple fiber components including cellulose, hemicellulose, pectin, dextrin, and resistant starch (RS). Approximately 15–20 g/day of RS is recommended as part of the diet, but most people eating a Western diet fail to achieve this level (1), consuming 4.9 g/day on average (2). Four major types of RS (RS1-4) have been defined based on their physical and chemical properties (1). Type 2 RS (RS2) is characterized by a compact granular structure that limits the accessibility of digestive enzymes and includes foods like resistant potato starch (RPS), raw banana starch, and high-amylose starch (1).

Consumption of RS, including RS2, has been shown to alter the microbiome and is associated with changes in short-chain fatty acid (SCFA) levels in the cecum and colon of rodents (3–6), cats (7), dogs (8), pigs (9–11), and humans (12, 13). Many of the potential health benefits of dietary fibers and RS are attributed to SCFA production by microbial fermentation (14). Each type of fiber and RS has the potential to differentially alter microbial composition which, in turn, can affect the production of bacterial metabolites (15). Not all members of one RS type have the same biological effects due to differences in their fine structure that may favor certain bacteria strains over others (16, 17). RS2 has been shown to alter the microbiome in rodents (4, 6, 18), pigs (19–22), and humans (13, 23–25).

Several studies have looked at the effect of RS on mice fed a high-fat diet (HFD). The microbiome of mice fed a HFD containing the RS2, high-amylose-maize starch (HAMS), was altered compared to those fed with the HFD only with decreasing Firmicutes and increasing Bacteroidetes (26). Mice fed a HFD had reduced alpha diversity compared to mice fed with a low-fat diet or those fed a HFD containing 10% HAMS or Fibersym RW (RS4) that also separated into four distinct groups in a principal component analysis (PCA) of β-diversity (27). Barouei et al. also observed changes to both alpha and beta diversity in mice fed with an HFD with 20% HAMS compared to mice fed with only the HFD (28). They also noted an increase in Bacteroidetes, a decrease in Firmicutes, and specific changes to the gene expression of pattern recognition receptors in both the ileum and the cecum due to RS2 feeding. The HFD used in these studies, however, does not resemble a typical Western diet. The percent of calories from fat was 45%, with lard being the major fat source and 8–19% sucrose by weight. The diets used in these studies do not reflect the type of diet consumed by many Americans (Western diet) which is low in certain micronutrients and contains fat from multiple sources, including highly saturated fat. In addition, these diets often lack a fermentable dietary substrate (29). Therefore, it is unclear if the observed changes would occur with a diet that more accurately reflected a typical American diet.

To address this question, we conducted studies using a rodent Total Western Diet (TWD) formulated using the 50th percentile daily intake levels for macro and micronutrients from the National Health and Nutrition Examination Survey (NHANES) which has fewer calories from protein and carbohydrates, double the fat including more saturated and monounsaturated fats, less polyunsaturated fat, and fewer complex carbohydrates, and two times the level of simple sugars compared to the AIN-93 G diet (30) that was supplemented with different levels of RPS. The TWD has been compared to the AIN-76A or AIN-93 diet in several studies. The TWD did not induce metabolic syndrome in mice (31). However, the abundance of aberrant crypt foci (ACF) in azoxymethane-initiated mice was nearly three times greater in mice fed with TWD compared to AIN-93G (32). Furthermore, consumption of green tea extract suppressed ACF development only in mice fed with the TWD. In addition, mice fed with the TWD developed more severe and prolonged dextran sulfate sodium (DSS)-induced colitis compared to AIN93G fed mice, ultimately leading to increased colon tumorigenesis (33). An additional study showed that walnut consumption, in the context of a TWD, mitigated (2.3-fold) azoxymethane-induced tumor production in mice, and the AIN-76A diet (1.3-fold) had less of an effect despite having a slightly larger amount of walnuts (34). Here, we show that feeding mice with the RPS-supplemented TWD led to dose-dependent changes in tissue morphology, the cecal microbiome, and gene expression in the cecum, proximal colon (PC), and distal colon (DC).

## Materials and Methods

### Animals and Diet

C57BL/6 mice were originally purchased from Charles River (Frederick, MD) and bred in-house. Mice were housed in ventilated filter-top cages at the United States Department of Agriculture-Beltville Human Nutrition Research Center (USDA BHNRC) animal facility under 12-h light/dark cycle. Timed breedings were set up and offspring were weaned at 3–4 weeks of age. Breeding pairs were fed rodent chow (Teklad 2020X, Frederick, MD). After weaning, mice were placed on the TWD (Supplementary Table 1, Envigo, Madison, WI) (30). After feeding mice with the TWD for 6 weeks, mice were divided into four dietary treatment groups: (1) TWD, (2) TWD supplemented with 2% w/w RPS (Ingredion, Westchester, IL), (3) TWD supplemented with 5% w/w RPS, or (4) TWD supplemented with 10% w/w RPS for an additional 3 weeks. Typically, potato starch is 50–70% RS2. In this study, the RPS levels chosen are approximately equivalent to a human consuming between 5 and 30 g (maximum range) of RS/day based on the nutrient density calculations used in formulating the TWD (energy density 4.4 kcal/g) (30), the NHANES 50th percentile caloric intake of 2,070 kcal/day, and potato starch typically containing 50–70% RS2. Body weights and food consumption were recorded weekly. All experiments were approved by the USDA-Agricultural Research Service (ARS) Beltsville Institutional Care and Use Committee.

### Sample Collection and Processing

Fecal samples, collected during the last 5 days, were flash-frozen and stored at −80°C. After 21 days on the four dietary treatments, mice were then euthanized. The colon and cecum were removed, a 1-cm segment of PC, 0.5 cm distal from the cecum was taken for RNA isolation before the terminal 6 cm of the colon was excised for fecal pellets to be removed and weighed. A section of the colon 0.5 cm from the anal terminus was taken for RNA isolation. The cecum was weighed. The cecal contents were then collected and sections of the cecum were taken for RNA isolation. Fecal pellets were weighed and homogenized in five volumes of water, centrifuged to removed debris, and the pH of the supernatant measured.

### Short-Chain Fatty Acid Analysis

Cecal contents were weighed and processed for SCFA analysis according to the method of Ward et al. with slight modifications (35). Distilled water and a metaphosphoric acid solution (250 g/L) containing 1 g/L of ethylbutyric acid as an internal standard were added according to the ratio of 1:9:2 (sample:water:acid/internal standard). Tubes were vortexed for 5 min and then centrifuged at 10,000 × *g* for 10 min. Approximately 200 ul of the supernatant was transferred to an insert in a gas chromatography vial.

An eight-point calibration was performed with a mix of six acids. Acetic, propionic, and butyric acids were present from 10 to 0.08 mM, and isobutyric, isovaleric, and valeric acids were present from 5 to 0.04 mM. To prepare the standards, 1 ml of each dilution was mixed with 0.2 ml of the metaphosphoric/ethylbutyric acid solution. Samples were analyzed by gas chromatography on a Shimadzu GC2010 equipped with a ZB-FFAP column (30 m × 0.52 mm ID × 1.0 μm film thickness; Phenomenex, Torrance, CA) and a flame ionization detector. The injector was maintained at 200°C, and 1 μl was injected at a split ratio of 5:1. The initial column temperature was 60°C and was held for 1 min. The column was heated at an increasing temperature of 17°C/min to 260°C and held for 8 min. Peaks were identified according to the retention time of individually run acids. For both external standards and the samples, raw peak areas were normalized to the ethylbutyric acid area. Peak area ratios were converted to concentration using regression equations for each acid. The overall concentration of acid in the sample was determined by multiplying the volume of water added by the concentration derived from the regression equation and dividing by the sample mass.

### 16S Sequencing of Cecal Contents

Deoxyribonucleic acid (DNA) was isolated from cecal contents using a *Quick*-DNA Fecal/Soil Microbe Microprep Kit (Zymo, Irvine, CA) following the manufacturer's instructions. The resulting samples were then further purified using the DNA Clean and Concentration kit (Zymo, Irvine, CA). The DNA concentration of the samples was determined using a Quant-it PicoGreen dsDNA kit (Invitrogen, Waltham, MA). DNA was submitted to the Michigan State University Research Technology Support Facility (RTSF) Genomics Core for targeted amplicon library preparation and sequencing. The V3-V4 hypervariable regions of the 16S rRNA gene were essentially amplified using the indexed Illumina compatible primers 341f/806r as described (36), except that the V3 flanking primer 341f was substituted for the V4 primer 515f as described in the paper. The pooled libraries were loaded into an Illumina MiSeq v2 500 cycle reagent cartridge. Custom sequencing and index primers complementary to the 341f/806r target-specific sequences were added to appropriate wells as previously described (36). The FASTQ files with raw data were submitted to the National Center for Biotechnology Information (NCBI) Sequence Read Archive (SRA) under the accession numbers PRJNA757013.

The 16S rRNA tag data curation and processing were performed using the CLC Microbial Genomics Module (QIAGEN Bioinformatics, Redwood City CA) following its standard Operational Taxonomic Unit (OTU) clustering workflow. Briefly, paired reads were first merged into contigs, followed by removal of the adapters, nucleotides below Q30, and reads containing more than two ambiguous nucleotides or shorter than five. Samples were then filtered by minimum reads of 100 or the minimum 50% from the median times the median number of reads across all samples. These processed contigs were subsequently aligned to the SILVA SSU database from release v138.1 (37). Chimeras were detected with k-mer search and removed from further processing and analysis. Sequences were then clustered into OTUs at 97% similarity. To estimate the alpha and beta diversity, OTUs were aligned with the MUSCLE tool (38) to reconstruct the phylogenetic tree by a Maximum Likelihood approach. The permutational multivariate ANOVA (PERMANOVA) (39) was performed to measure the effect size and significance of beta diversity. OTU count abundance was further collapsed into different taxon levels by the JMP Genomics (SAS, Cary, NC). Successively, PCA and hierarchical clustering were performed with the OTU or taxon-specific abundance profiles to examine the changes induced by the RPS treatments. Data distribution induced by the RPS treatments was visualized by PCA in JMP Genomics 10 with default settings. Additionally, the abundance profiles were subjected to linear discriminant analysis effect size [LEfSe (40), an analysis to identify RPS-specific biomarkers at multiple taxonomical levels]. The differential expression analysis for all taxa and OTUs was performed with the Bioconductor package DEseq2 (41) (version 3.14; run on RStudio, version 4.0.3, Boston, MA).

### RNASeq Analysis of Cecum, Proximal, and Distal Colon Tissue

RNA from the cecum, PC, and DC were isolated using the Tri-Reagent (Zymo, Irvine, CA) and the Purelink RNA kits (Invitrogen, Carlsbad, CA) and further purified using the RNA Clean and Concentrate columns (Zymo, Irvine, CA). RNA was submitted to the Michigan State University RTSF Genomics Core facility for sequencing. Libraries were prepared using the Illumina TruSeq Stranded mRNA Prep kit with Integrated DNA Technologies (IDTs) for Illumina TruSeq RNA Unique Dual (UD) Indexes following the manufacturer's recommendations. Sequencing of the cDNA Libraries was performed using the Illumina HiSeq 4,000 single-read flow cell in a 1 × 50 bp single-end format using the HiSeq 4,000 sodium bisulfate (SBS) reagents. The FASTQ files with raw data and the gene expression profiles were submitted to the NCBI Gene Expression Omnibus (GEO) under the accession numbers GSE182458.

The resulting sequences were processed to determine the gene expression levels as follows.

Nucleotides below Q30 or reads containing more than two ambiguous nucleotides were removed before sequence alignments performed by the CLC Genomics Workbench version 20.01 (QIAGEN Bioinformatics, Redwood City CA). To calculate the gene expression in the count, reads were mapped to the Mus musculus genome assembly GRCm39. Transcriptomes built from the alignment results were subjected to differential expression analysis. The statistical analyses were carried out with DEseq2. Genes were considered differentially expressed with the thresholds of a false discovery rate (FDR) ≤.05 and an absolute fold change ≥ 1.5. We functionally annotated the differentially expressed genes using our Porcine Translational Research Database (42) as previously described (43). The database serves to translate data found in rodents or pigs to humans. We also assigned cell specificity to differentially expressed genes using the database. Venn analysis was conducted using the online tool Venny 2.1 (https://bioinfogp.cnb.csic.es/tools/venny/).

## Results

### Effect of RPS on Short-Chain Fatty Acid Production, Tissue Weights, and Fecal pH

**Figure 1:**
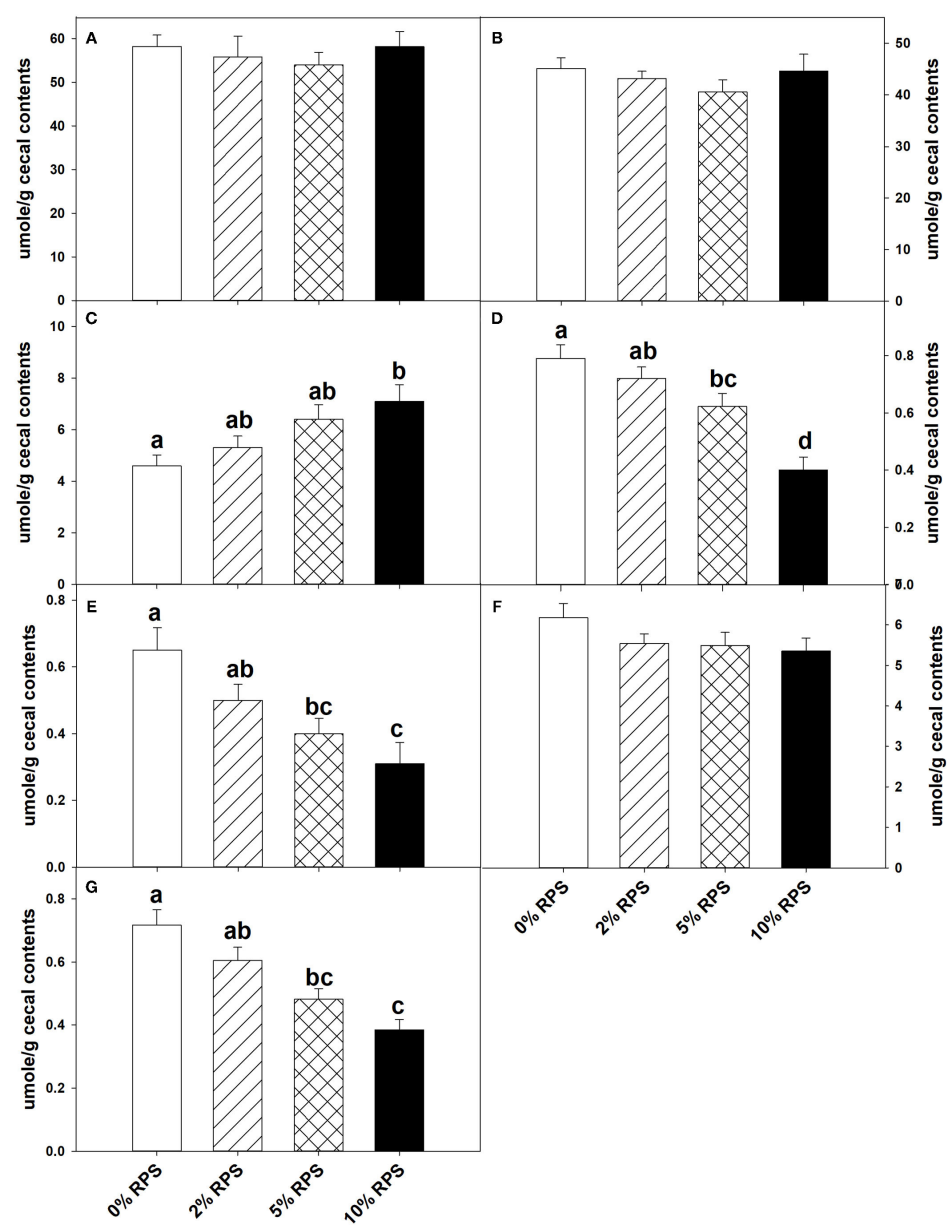
Feeding mice resistant potato starch (RPS) alters cecal short-chain fatty acid (SCFA) production. Mice were fed the basal Total Western Diet (TWD) for 6 weeks. Subsets of mice continued to receive the TWD or were fed the TWD plus 2, 5, or 10% RPS for an additional 3 weeks before the cecal contents were collected for short-chain fatty acids (SCFA) analysis as described in the material and methods section. Data is from two replicate experiments and are expressed as Mean ± SEM, *n* = 14–18 mice/group. **(A)** Total SCFA, **(B)** Acetic acid, **(C)** Butyric acid, **(D)** Isobutyric acid, **(E)** Isovaleric acid, **(F)** Propionic acid, and **(G)** Valeric acid. Groups with different letters are significantly different, *p* < 0.05, by ANOVA (Holm-Sidak).

At weaning the mice were placed on the TWD for six weeks prior to switching to the RPS-containing diets to allow sufficient time for the effects of feeding the TWD on metabolism, growth rate and microbiome changes induced by weaning to stabilize (44). Subsets of mice were then fed with the TWD containing 0, 2, 5, or 10% RPS for an additional 3 weeks before being euthanized to collect tissues for analysis. Feeding different levels of resistant starch diets for 3 weeks had no effect on weight gain or feed consumption (Supplementary Figure 1A and data not shown). It has been reported that feeding RPS to mice can result in an increase in colon and cecum weight (45, 46). We found that % colon/BW ratio increased with RPS consumption (Supplementary Figure 1C). Similarly, the %cecum/BW ratio increased with increasing levels of dietary RPS (Supplementary Figure 1B). Fermentation of resistant starches has been shown to increase the production of SCFAs (3–6). To verify that increased fermentation of RPS was occurring in the context of a TWD, we measured the SCFA levels in cecal contents of TWD and TWD/RPS-fed mice. Total SCFA levels and levels of acetate and propionate were not altered by the RPS diets (Figures 1A,B,F). The butyric acid levels, however, increased with increasing dietary RPS, but only achieved significance in mice fed with the 10% RPS diet (Figure 1C). The levels of isobutyric and isovaleric acid decreased (Figures 1D,E), and we found that fecal pH decreased with increasing dietary RPS (Supplementary Figure 2).

### Effect of RPS on the Microbiome Diversity and Composition

**Figure 2:**
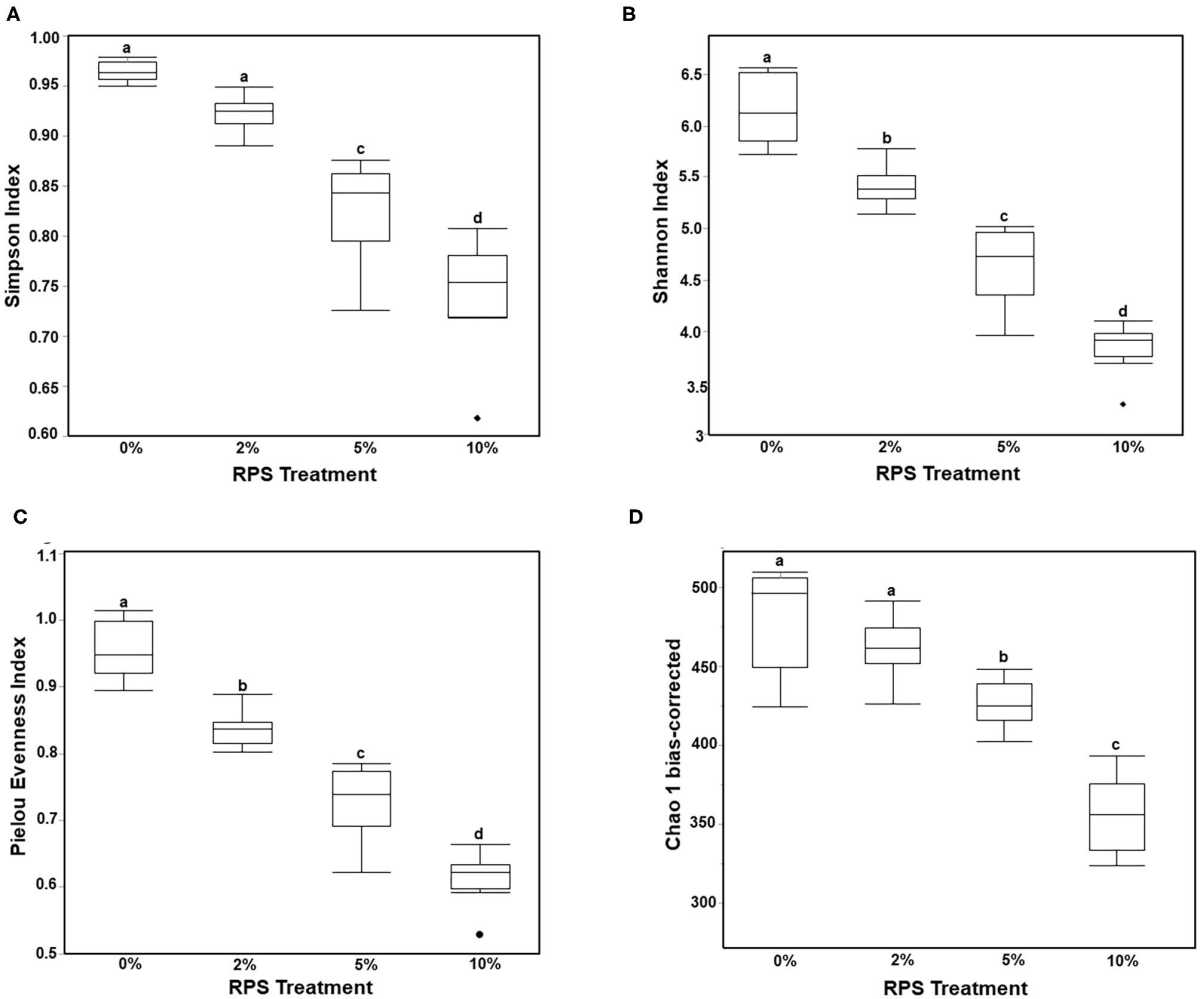
Feeding mice increasing levels of RPS leads to decreased microbiome diversity. The cecal microbiome of mice fed the TWD or TWD 2, 5, or 10% RPS was analyzed by 16S sequencing as described in the material and methods section. Simpson Index of Diversity **(A)**, Shannon Diversity Index **(B)**, the Pielou Evenness Index **(C)**, and the Chao-1 biased Index **(D)** plots were generated using the JMP Genomics program and analyzed by One-way ANOVA with Tukey-Kramer *post-hoc* analysis. Groups with different letters are significantly different, *p* < 0.05 *n* = 8–9 mice/group.

**Figure 3:**
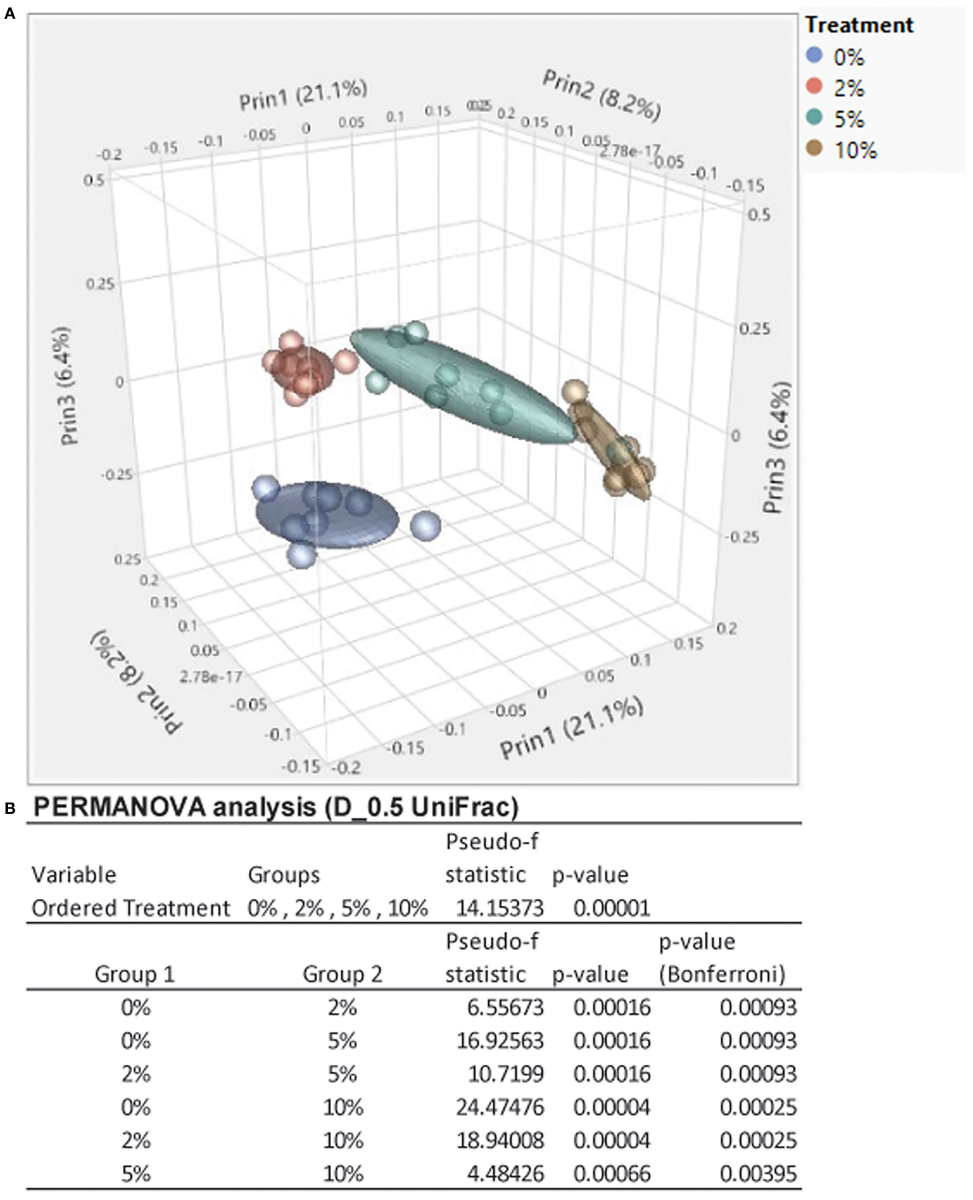
Feeding mice increasing levels of RPS leads to clear separation by diet in a principal component analysis (PCA) plot and permutational multivariate ANOVA (PERMANOVA) analysis. **(A)** A PCA plot was generated using JMP Genomics and shows distinct clustering of mice by dietary group with mice in the TWD cluster separated away from the three clusters of mice fed different levels of RPS. **(B)** Results of a 0.5 UniFrac PERMANOVA analysis performed using CLC Genomics. *n* = 8–9 mice/group.

Consumption of different fibers and resistant starches can differentially alter the microbiome (12, 47). To see how consumption of RPS affected the microbiome in the context of a TWD, we performed 16S sequencing on cecal contents from male mice fed with the TWD or TWD/RPS diets. Microbiome diversity was measured using the Simpson Index of Diversity, the Shannon Diversity Index, the Chao-1 biased index, and the Pielou Evenness Index which measure the different aspects of diversity including diversity, abundance, and evenness of species between dietary groups (Figure 2). All four indices showed a similar trend, with higher levels of RPS resulting in decreased species diversity, abundance, and evenness, indicating that consumption of RPS significantly affected the microbiome. B-diversity was significantly altered in an RPS-dependent manner with clear separation of the different dietary RPS groups by a PCA and PERMANOVA analysis (0.5 UniFrac) (Figure 3). Furthermore, mice fed with the three RPS diets clustered closer together and away from mice fed with the basal TWD. Similar results were obtained for a PERMAOVA analysis using the Bray-Curtis and Jaccard methods (data not shown). At the phylum level, Actinobacteria significantly increased from 0.1% to ~1.1%, and for the two major phyla, Bacteroidetes and Firmicutes, there was a trend toward decreasing Bacteroidetes and increasing Firmicutes (data not shown).

**Figure 4:**
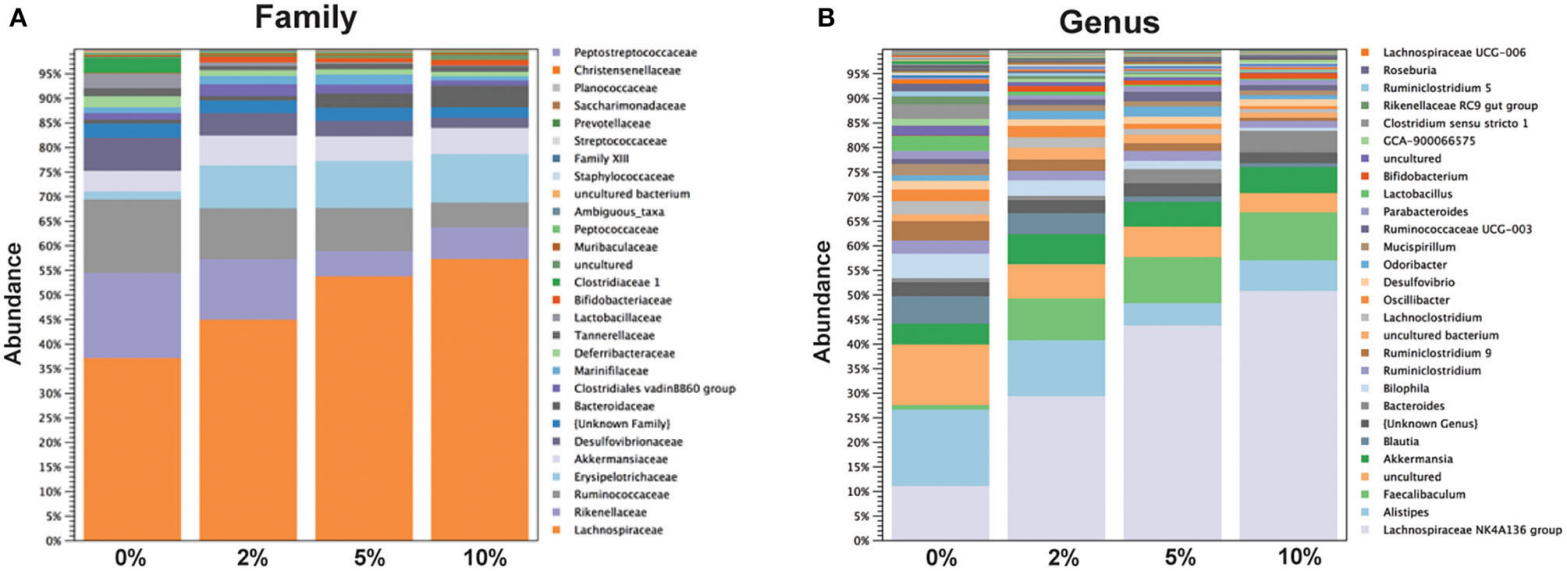
Feeding mice increasing levels of RPS led to alterations in relative abundance of taxa at both the family and genus level. Stack plots showing the relative abundance of taxa at the **(A)** family and **(B)** genus level. Stack plots were generated using CLC Genomics and relative abundance data, *n* = 8–9 mice/group.

**Figure 5:**
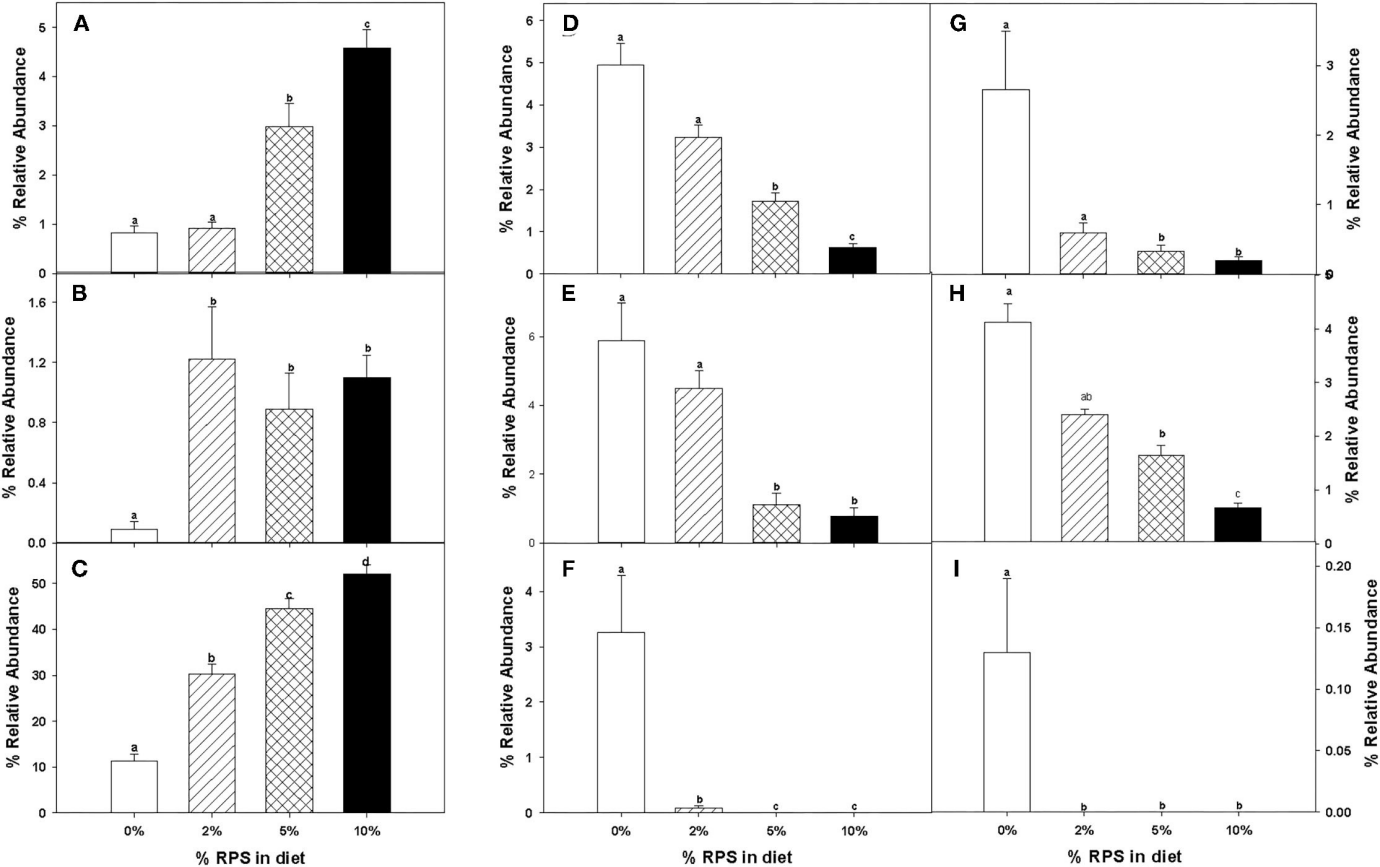
The relative abundance of specific taxa was altered by feeding mice different levels of RPS. The effect of feeding different levels of RPS on the relative abundance of specific taxa is shown. **(A)** *Bacteroides*, **(B)** *Bifidobacterium* ambiguous taxa, **(C)** *Lachnospiraceae NK40A136* group, **(D)** *Bilophia*, **(E)** *Blauta*, **(F)** *Clostridium sensu stricto 1*, **(G)** *Lactobacillus*, **(H)** *Ruminiclostridium 9*, and **(I)** *Turicibacter*. Data are the Mean ± SEM, *n* = 8–9 mice/group. Bars with different letters are significantly different *p* < 0.05.

The relative abundance of various families is shown in Figure 4A and Table 1. *Lachnospiraceae* was the dominant family, and its abundance increased with dietary RPS levels. The abundance of both *Rikenellaceae* and *Ruminococcaceae* decreased with increasing dietary RPS, as did *Clostridiaceae 1, Deferribacteraceae, Desulfovibrionaceae*, Family XIII, and *Lactobacillaceae*. Several families saw their abundance increase with increasing dietary RPS, including *Bacteroidaceae, Bifidobacteriaceae*, and *Erysipelotrichaceae*. Similar shifts were observed at the genus level (Figure 4B and Table 2). The abundance of *Lachnospiraceae NK4A136* group dramatically increased with increasing RPS in the diet, becoming the dominant genus at higher RPS levels occupying ~50% of the microbiome (Figure 5C and Table 2). Other genera that also increased in response to dietary RPS included *Bacteroides* and *Bifidobacterium* ambiguous taxa (Figures 5A,B). *Bifidobacterium* abundance increased significantly at the lowest level of RPS tested (2%) but did not increase further at higher RPS levels. This contrasts with the Bacteroides and *Lachnospiraceae NK4A136* groups which increased in abundance with increasing dietary RPS levels. As indicated by the decrease in alpha-diversity, many more genera decreased than increased in abundance in response to dietary RPS. These included *Alistripes, Bilophila, Balutia, Clostridium sensu stricto 1, GCA-900066575, Lachnoclostridium, Lactobacillus, Lactobacillus, Ambiguous taxa, Rikenellaceae RC9 gut group Ambiguous taxa, Roseburia, Ruminiclostridium 5* and *9, Ruminococcaceae UCG-014, Ruminococcaceae UCG-014 Ambiguous taxa*, and *Turicibacter* (Figures 5D–I and Table 2). Four genera, *Clostridium sensu stricto 1, Lactobacillus Ambiguous taxa, Ruminococcaceae UCG-014* A*mbiguous taxa*, and *Turicibacter* had their abundance fall to very low or undetectable levels.

**Table 1:** Differential expression of families with a relative abundance of at least 0.05% in microbiota isolated from mice fed different levels of dietary RPS.

	**Level of dietary RPS**							
	**0%**		**2%**		**5%**		**10%**	
	**Mean^1^**	**SEM**	**Mean**	**SEM**	**Mean**	**SEM**	**Mean**	**SEM**
Akkermansiaceae	4.25^a^	0.93	6.02^a^	1.15	5.16^a^	0.55	5.39^a^	0.58
Bacteroidaceae	0.82^a^	0.14	0.92^a^	0.12	2.98^b^	0.47	4.54^b^	0.37
Bifidobacteriaceae	0.09^a^	0.05	1.22^b^	0.35	0.89^b^	0.24	1.16^b^	0.14
Clostridiaceae 1	3.28^a^	1.04	0.09^b^	0.04	0.00^c^	0.00	0.00^c^	0.00
Clostridiales vadinBB60 group	1.47^a^	0.52	2.43^a^	0.25	1.89^a^	0.26	1.05^a^	0.17
Deferribacteraceae	2.36^a^	0.51	1.27^b^	0.17	1.10^b^	0.15	1.00^b^	0.18
Desulfovibrionaceae	6.66^a^	0.65	4.66^b^	0.39	3.20^bc^	0.30	2.03^c^	0.24
Erysipelotrichaceae	1.73^a^	0.67	8.83^b^	1.54	9.85^b^	1.38	10.16^b^	1.37
Family XIII	0.07^a^	0.01	0.03^b^	0.00	0.02^b^	0.00	0.01^c^	0.00
Lachnospiraceae	38.50^a^	1.79	46.66^ab^	2.84	55.15^bc^	1.67	58.64^c^	1.98
Lactobacillaceae	3.19^a^	1.03	0.80^b^	0.25	0.34^bc^	0.09	0.20^c^	0.06
Marinifilaceae	1.19^ac^	0.17	1.71^bc^	0.19	2.10^b^	0.28	0.85^a^	0.14
Muribaculaceae	0.42^a^	0.05	0.46^a^	0.04	0.41^a^	0.05	0.43^a^	0.05
Peptococcaceae	0.31^a^	0.04	0.29^a^	0.03	0.24^a^	0.02	0.24^a^	0.02
Rikenellaceae	17.62^a^	1.08	12.61^a^	0.51	5.43^b^	0.38	6.67^b^	0.85
Ruminococcaceae	15.52^a^	0.60	10.86^b^	0.55	9.20^b^	0.92	5.13^c^	0.23
Staphylococcaceae	0.07^a^	0.03	0.01^b^	0.00	0.03^ab^	0.01	0.04^a^	0.02
Tannerellaceae	1.68^a^	0.47	0.80^a^	0.13	1.21^a^	0.17	1.12^a^	0.39

1 *Values are the Mean ± SEM of normalized relative abundance values, n = 8–9*.

*Values with different letters are signficantly different, adjusted p < 0.05*.

**Table 2:** Differential expression of genera with a relative abundance of at least 0.05% in microbiota isolated from mice fed different levels of dietary RPS.

	**Level of dietary RPS**							
	**0%**		**2%**		**5%**		**10%**	
	**Mean^1^**	**SEM**	**Mean**	**SEM**	**Mean**	**SEM**	**Mean**	**SEM**
Acetatifactor, Ambiguous_taxa	0.08^ab^	0.02	0.08^a^	0.01	0.03^b^	0.01	0.01^b^	0.00
Akkermansia	4.25^a^	0.93	6.02^ab^	1.15	5.17^ab^	0.55	5.43^b^	0.59
Alistipes	14.4^a^	0.74	10.35^a^	0.48	3.74^b^	0.47	5.61^a^	0.80
Alistipes, Ambiguous_taxa"	1.39^a^	0.31	1.30^a^	0.21	1.01^a^	0.20	0.89^a^	0.16
Azospirillum sp. 47_25	0.19^a^	0.05	0.12^a^	0.01	0.31^b^	0.06	0.46^b^	0.09
Bacteroides	0.82^a^	0.14	0.92^a^	0.12	2.99^b^	0.47	4.57^c^	0.38
Bifidobacterium, Ambiguous_taxa	0.09^a^	0.05	1.22^b^	0.35	0.89^b^	0.24	1.17^b^	0.15
Bilophila	4.95^a^	0.50	3.23^a^	0.29	1.72^b^	0.19	0.63^c^	0.08
Blautia	5.90^a^	1.10	4.50^a^	0.51	1.12^b^	0.33	0.77^b^	0.26
Catenibacterium	0.53^a^	0.29	0.15^a^	0.04	0.14^a^	0.04	0.09^a^	0.02
Clostridium sensu stricto 1	3.26^a^	1.03	0.09^b^	0.04	0.00^c^	0.00	0.00^c^	0.00
Desulfovibrio	1.70^a^	0.29	1.42^a^	0.15	1.49^ab^	0.18	1.42^b^	0.25
Dubosiella	0.08^a^	0.04	0.03^a^	0.01	0.03^a^	0.01	0.03^a^	0.01
Eisenbergiella	0.14^a^	0.05	0.02^a^	0.01	0.00^b^	0.00	0.00^b^	0.00
Faecalibaculum	0.99^a^	0.36	8.65^b^	1.53	9.69^b^	1.35	10.10^b^	1.35
GCA-900066575	1.55^a^	0.34	0.70^b^	0.06	0.70^ab^	0.10	0.35^b^	0.05
Harryflintia	0.05^a^	0.01	0.05^a^	0.01	0.04^a^	0.01	0.03^a^	0.00
Intestinimonas	0.10^a^	0.03	0.11^a^	0.02	0.13^a^	0.04	0.03^a^	0.02
Lachnoclostridium	2.22^a^	0.17	1.62^a^	0.13	0.86^b^	0.08	0.58^b^	0.07
Lachnoclostridium, Ambiguous_taxa	0.64^ab^	0.19	0.64^a^	0.09	0.36^ab^	0.05	0.18^b^	0.02
Lachnospiraceae FCS020 group	0.23^a^	0.03	0.19^a^	0.03	0.15^a^	0.02	0.12^a^	0.02
Lachnospiraceae NK4A136 group	11.36^a^	1.47	30.21^b^	2.25	44.53^c^	2.18	52.06^d^	2.02
Lachnospiraceae NK4A136 group, Ambiguous_taxa	0.08^a^	0.01	0.10^a^	0.01	0.16^b^	0.01	0.18^b^	0.01
Lachnospiraceae UCG-001	0.09^a^	0.04	0.13^a^	0.03	0.06^a^	0.01	0.01^b^	0.00
Lachnospiraceae UCG-006	0.86^a^	0.26	0.38^a^	0.06	0.27^a^	0.04	0.37^a^	0.10
Lachnospiraceae UCG-008	0.57^a^	0.06	0.17^b^	0.04	0.02^c^	0.01	0.01^c^	0.00
Lactobacillus	2.65^a^	0.85	0.59^b^	0.15	0.33^b^	0.09	0.19^b^	0.06
Lactobacillus, Ambiguous_taxa	0.55^a^	0.22	0.22^b^	0.12	0.01^c^	0.00	0.00^c^	0.00
Mucispirillum	2.37^a^	0.51	1.27^a^	0.17	1.10^a^	0.15	1.01^a^	0.18
Odoribacter	1.19^a^	0.17	1.71^bc^	0.19	2.11^b^	0.28	0.85^ac^	0.14
Oscillibacter	2.40^ab^	0.25	2.33^a^	0.26	1.16^ab^	0.21	0.67^b^	0.13
Parabacteroides, Ambiguous_taxa	1.68^ab^	0.47	0.81^a^	0.13	1.21^ab^	0.17	1.13^b^	0.40
Peptococcus	0.14^a^	0.04	0.18^a^	0.02	0.13^a^	0.02	0.10^a^	0.02
Rikenella	0.25^ab^	0.02	0.41^a^	0.05	0.38^a^	0.07	0.07^b^	0.03
Rikenellaceae RC9 gut group, Ambiguous_taxa	1.56^a^	0.41	0.52^b^	0.06	0.28^bc^	0.05	0.13^c^	0.04
Roseburia	1.56^a^	0.50	0.36^b^	0.12	0.14^b^	0.06	0.019^c^	0.00
Ruminiclostridium	2.85^a^	0.34	2.07^a^	0.11	2.17^a^	0.22	1.48^a^	0.08
Ruminiclostridium 5	1.09^a^	0.16	0.66^a^	0.07	0.30^b^	0.03	0.20^b^	0.02
Ruminiclostridium 9	4.12^a^	0.35	2.46^ab^	0.11	1.64^b^	0.19	0.67^c^	0.08
Ruminococcaceae NK4A214 group	0.08^a^	0.02	0.13^ab^	0.01	0.14^b^	0.02	0.14^b^	0.03
Ruminococcaceae UCG-003	1.11^a^	0.24	1.22^ab^	0.33	2.01^b^	0.45	1.13^b^	0.10
Ruminococcaceae UCG-004	0.22^a^	0.02	0.17^a^	0.01	0.10^ab^	0.02	0.02^b^	0.01
Ruminococcaceae UCG-009	0.15^a^	0.04	0.11^a^	0.01	0.09^a^	0.01	0.08^a^	0.01
Ruminococcaceae UCG-014	0.25^a^	0.06	0.29^a^	0.06	0.27^a^	0.10	0.07^a^	0.03
Ruminococcaceae UCG-014, Ambiguous_taxa	0.25^a^	0.08	0.12^a^	0.04	0.00^b^	0.00	0.00^b^	0.00
Ruminococcus 1	0.46^ab^	0.14	0.20^a^	0.02	0.33^b^	0.05	0.31^b^	0.07
Staphylococcus	0.07^a^	0.03	0.01^b^	0.00	0.032^ab^	0.01	0.04^a^	0.02
Subdoligranulum	0.23^a^	0.07	0.04^b^	0.01	0.10^ab^	0.04	0.06^ab^	0.03
Turicibacter	0.13^a^	0.06	0.00^b^	0.00	0.00^b^	0.00	0.00^b^	0.00
Tyzzerella	0.39^ab^	0.05	0.26^a^	0.03	0.27^ab^	0.05	0.28^b^	0.05

1 *Values are the Mean ± SEM, n = 8–9*.

*Values with different letters are signficantly different, adjusted p < 0.05*.

**Figure 6:**
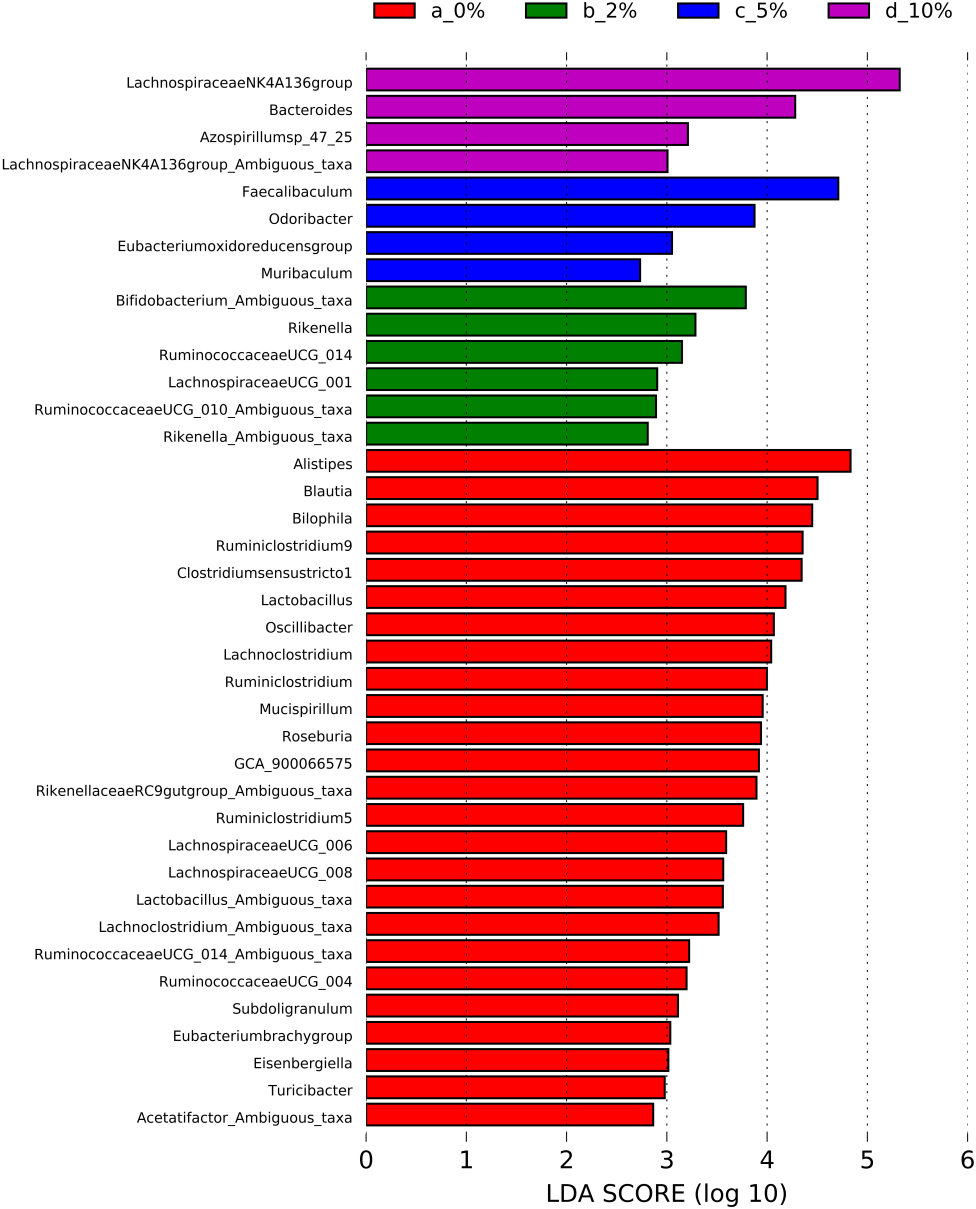
Linear discriminant analysis effect size (LEfSe) analysis identifies differentially abundant genera as biomarkers. 16S sequencing data from the cecal microbiome of mice fed the TWD or TWD plus 2, 5 or 10% RPS were analyzed by the LEfSe method using the Kruskal-Wallis test (*p* < 0.05) with linear discriminant analysis (LDA) score > 2.0 being considered significant. *n* = 8–9 mice/group.

To identify bacterial genera that discriminate between consumption of different levels of dietary RPS, a Lefse plot was generated for Family (Supplementary Figure 3) and Genera taxa (Figure 6). The all-group LefSe comparison showed that *Erysipelotrichaceae* and *Bifidobacteriaceae* were the top two discriminating families associated with mice fed with a 10% RPS diet, and both had their relative abundance increased with increasing levels of dietary RPS. No families that were discriminating for mice fed with the 5% RPS diet were identified at the family level, while *Ruminococcaceae* was most discriminating for mice fed with a 2% RPS diet. In addition, *Rikenellaceae* and *Desulfovibrionaceae* both had their relative abundance decreased with increasing levels of RPS. *Lachnospiraceae NK4136 group* and *Bacteroidetes* were the top two genera that were differentially associated with mice fed with the 10% RPS diet. These genera increased in mice fed with diets containing RPS. The top two discriminating genera for mice fed with the 5% RPS diet were *Faecalibaculum* and *Odoribacter*. *Bifidobacterium amibiguous taxa* and *Rikenella* were the two discriminating genera associated with mice fed with the 2% RPS. Genera associated with consumption of the basal TWD compared to RPS-containing diets included *Alistripes, Blauta, Bilophia, Clostridium sensu stricto 1, Lactobacillus*, and *Ruminiclostridium 9* which all had their relative abundance decreased by increasing RPS consumption (Table 2). The defining characteristic of the microbiome of mice fed with increasing amounts of RPS is the decreased abundance of a large number of genera and the domination of the microbiome by the *Lachnospiraceae NK4A136* group especially in mice fed with the 5 and 10% RPS diets.

### Effect of RPS on Gene Expression in the Cecum, PC, and DC

**Figure 7:**
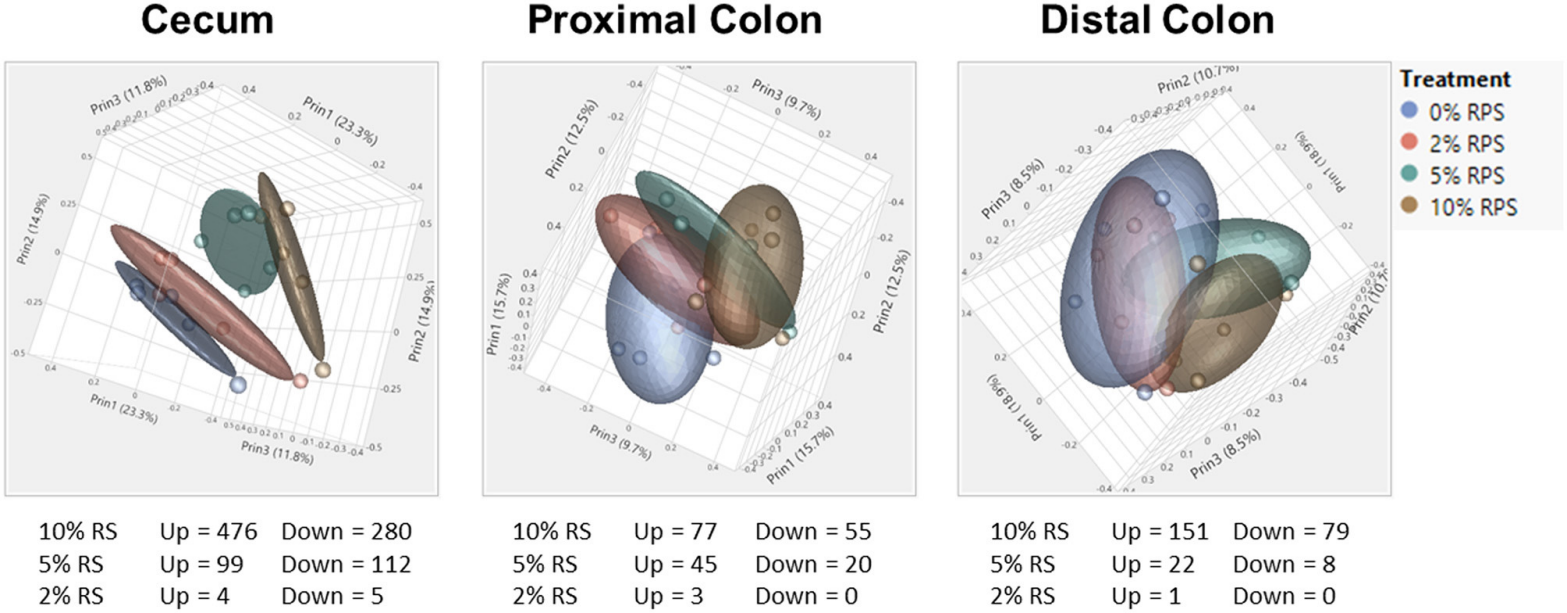
PCA of RNAseq data from the cecum, proximal, and distal colon. PCA plots were generated from RNAseq data for the cecum, distal, and proximal colon. In the cecum, the four dietary groups were well-separated but the degree of separation decreased gradually in the proximal and distal colons by distance. Plots were generated using JMP Genomics. *n* = 8–9 mice/group.

The full RNASeq analysis for the cecum, PC, and DC can be found in Supplementary Tables 2–4, respectively. In the analysis of differentially expressed genes in three tissues (cecum, PC, and DC) and four treatment groups (0% RPS, 2% RPS, 5% RPS, and 10% RPS), there were 1101 genes that were upregulated or downregulated >1.5-fold at an FDR adjusted *p* < 0.05 compared to each retrospective control group. These genes formed four distinct clusters by PCA in the cecum, but the trend was less evident in the PC and even less so for DC (Figure 7). Despite these qualitative differences, the proportion of variance in gene expression due to treatment in each tissue was the same, 13% in cecum and PC and 12% in the DC (data not shown). The number of upregulated genes by 10% RPS in the cecum, PC, and DC was 476, 77, and 151 genes, respectively.

The top 20 differentially expressed genes in the cecum, PC, and DC, respectively, are shown in Tables 3–**5**. The number of genes downregulated by 10% RPS in the cecum, PC, DC was 280, 55, and 79, respectively. Animals fed with 5% RPS had 211 genes that were differentially upregulated (99) or downregulated (112) by more than 1.5-fold in the cecum. Four genes were upregulated and five were downregulated by 2% RPS in the cecum. Forty-five genes were upregulated, and 20 genes were downregulated by 5% RPS in the PC. Three genes were upregulated, and no genes were downregulated by 2% RPS in the PC. Twenty-two genes were upregulated and 8 were downregulated by 5% RPS in the DC. Only gene was upregulated by 2% RPS in the DC.

**Table 3:** Top 20 differentuially expressed genes in the cecum.

**Gene**	**2 vs. 0%**		**5 vs. 0%**		**10 vs. 0%**	
	**FC**	***p*** **(adj)**	**FC**	***p*** **(adj)**	**FC**	***p*** **(adj)**
Cyp24a1					−25.8	3.98E-05
Apoa4	−3.7	5.75E-04	−14.6	1.93E-15	−20.7	3.61E-17
Psca			−5.3	9.19E-03	−17.0	7.42E-06
Ly6g			−23.3	4.86E-05	−11.9	7.35E-04
Cpn2					−11.9	1.03E-02
Ighv1-85			−17.8	3.02E-02	−8.9	6.99E-02
ALPG					−8.0	4.34E-03
Mfsd2a			−6.8	1.09E-03	−7.1	2.06E-04
Abcb11			−7.0	1.73E-02	−7.1	6.35E-03
Prss22			−11.1	3.01E-04	−6.4	2.29E-03
Mmp10			−4.7	5.92E-02	−6.2	6.78E-03
Sema7a			−3.1	1.22E-07	−5.9	1.13E-20
B3galt5			−4.6	7.84E-06	−5.9	5.82E-09
Olr1	−2.9	1.19E-03	−3.8	1.07E-07	−5.9	4.48E-14
Gjb4			−2.7	5.32E-02	−5.7	3.48E-05
Il1rn			−4.3	5.54E-04	−5.7	2.26E-06
Scnn1g					−5.3	3.98E-05
Spam1					−5.2	4.62E-02
1700057G04Rik			−2.4	9.47E-03	−5.2	3.58E-10
Gm5155					−5.0	4.96E-02
Gja5			2.4	7.10E-03	4.7	2.19E-09
Igkv4-58					4.9	1.68E-02
Ighv1-34					4.9	3.03E-02
Igfbp2			2.6	1.41E-02	5.2	3.47E-08
Igkv13-84					5.4	4.96E-02
Ighv1-58			5.2	3.56E-02	5.6	8.94E-03
Igkv10-96					6.0	2.91E-02
Igkv6-13					6.1	3.28E-02
Krt17			2.3	2.55E-02	6.3	1.58E-11
Cyp4f15			3.0	2.76E-06	6.3	4.58E-20
Igkv6-32					6.3	3.47E-02
Nos2			2.9	3.67E-02	6.5	4.23E-07
Duoxa2			4.4	1.14E-02	6.7	8.27E-05
Ighg1			4.9	9.98E-03	7.0	1.45E-04
Ighv10-3					7.2	3.37E-04
Ighv2-2					9.8	1.86E-02
Cacng8	12.0	8.44E-02	9.8	1.46E-02	9.9	4.98E-03
Ighv1-63					10.0	1.41E-04
Ighv9-2					16.5	7.64E-03
Olfr1459			10.7	5.89E-02	18.9	2.67E-03
Ighv1-74			8.5	6.63E-02	19.7	8.66E-04

Five genes were upregulated in all three tissues by 10% RPS vs. control (Supplementary Figure 4). Ten genes were upregulated in the cecum and PC but not in the DC by 10% RPS. Sixteen genes were upregulated in the cecum and DC but not in the PC. Six genes were upregulated in the PC and DC but not in the cecum. In contrast, no genes were commonly downregulated in the cecum, PC, and DC. Five genes were downregulated in the cecum and DC but not in the PC. Three genes were downregulated in the cecum and PC but not in the DC. Lastly, two genes were downregulated in the PC and DC but not in the cecum.

Differentially expressed genes were functionally classified into two broad categories (genes related to Immunology or Metabolism) and further subdivided into specific functional categories. The fully classified datasets for cecum, PC, and DC are found in Supplementary Tables 5, 6 (cecum), Supplementary Tables 7, 8 (PC), and Supplementary Tables 9, 10 (DC). Selected pathways were extracted for emphasis and are found in **Tables 6**–**8** for cecum, PC, and DC, respectively.

## Discussion

The results presented here demonstrate that consumption of RPS in mice fed with a diet that emulates a typical American diet for both macro and micronutrients (30) and has significant effects on both the microbiome and gene expression profiles in the cecum, PC, and DC. The effects were dependent on the level of RPS in the basal TWD. The levels of RPS consumed by the mice are approximately equivalent to a human consuming a range between 5 and 30 g of RS/day depending on the percentage of RS2 in the potato starch (30). While significant changes to the microbiome occurred in mice fed with the 2% RPS TWD (e.g., *Bifidobacterium, Lachnospiraceae NK4A136 group*, and *Clostridium sensu stricto 1*), more genera were significantly altered in mice fed with the 5 or 10% RPS diets. The most significant change to the microbiome due to dietary RPS was the dramatic expansion of the *Lachnospiraceae NK4A136 group* which went from ~10 to 50% relative abundance in a graded manner as the dietary RPS levels went from 0 to 10%. Only three genera in addition to *Lachnospiraceae NK4A136 group* had their relative abundance increased (*Bacteroides, Bifidobacterium*, and *Faecalibaculum*). As a result of the large expansion of the *Lachnospiraceae NK4A136 group*, many other genera had their relative abundance decreased or were unchanged.

The *Lachnospiraceae* and *Ruminococcaceae* families contain the largest number of butyrate producers (48), and the *Lachnospiraceae NK4A136 group* contains members that can produce butyrate. The observed RPS-dependent increase in butyrate production coincides with the RPS-dependent increase in relative abundance of the *Lachnospiraceae NK4A136 group*, suggesting that the increase in butyrate production is likely due in part to the large increase in this genus, although some members of the *Bacteroides, Bifidobacterium*, and *Faecalibaculum* genera, all of which increased to a much lesser extent, contain potential butyrate producers that may have contributed as well. The role of these genera in butyrate production is further supported by the fact that several other genera, thought to be butyrate producers, including *Roseburia* and genera from the family *Ruminococcaceae* had decreased relative abundance with increasing dietary RPS, indicating that the observed increase in butyrate production cannot be due to these genera.

We found that feeding mice with RPS led to a decrease in α-diversity. Several other studies also showed decreased α-diversity in rodents fed with RS (6, 28, 49–51). Pigs fed with diets with added RS2, including RPS, also showed a decrease in α-diversity in the colon but not the cecum (19, 21). Reduced α-diversity was also observed in pigs fed with a diet containing RS3 (52). Similarly, we saw an increase in butyrate production and decreases in branched-chain SCFAs, indicative of a shift to saccharolytic fermentation and a decrease in proteolytic fermentation (branched-chain SCFAs) (53, 54). Consumption of resistant starches has also been associated with a decrease in fecal pH (5, 6) which can favor butyrate forming taxa (55). Others have also reported increased butyrate (5, 6, 56), and enlargement of the cecum in rodent studies (28, 45, 46). Decreased fecal pH (20, 57, 58), increased butyrate production, reduced levels of branched-chain SCFAs (9, 11, 58–60), and increased colon and cecum weight have also been observed in pigs fed with RS (11, 57), likely the result of increased levels of undigested fiber/RS reaching the cecum.

Changes in the microbiome taxa observed in response to feeding RS2 are variable between species. We found that mice fed with RPS in the context of a TWD (30) resulted in a large increase in *Lachnospiraceae NK4A136 group*; more modest increases in *Bacteroides, Bificobacterium*, and *Faecalibacterium*, and a decrease in the families *Clorstridiaceae, Ruminoccocaceae*, and *Roseburia spp*. Others reported different results with increases in members of the *Clorstridiaceae* and *Ruminoccocaceae* families that were, to some extent, either species or sampling location (feces, colon, or cecum)-dependent (61–63). Interestingly, one study found that mice either had similar *Lachnospiraceae* relative abundance to ours or high relative abundance of *Ruminoccocaceae* but not both (56), suggesting that there may be competition between and among members of these bacterial families for the RS. It has also been reported that a human subject's ability to respond to an RS diet is dependent on the baseline composition and abundance of specific bacterial species (64). In the basal TWD-fed mice, the relative abundance of *Lachnospiraceae*, and specifically *Lachnospiraceae NK4A136 group*, was substantially higher than many other groups and, when given access to RPS, may have had been able to outcompete the other taxa. Also, it has been reported that *Ruminococcus bromii* is dependent on a source of branched-chain fatty acids (BCFAs) for growth (65), and the decrease in these taxa in our studies may be related to the decrease in BCFA formation. In general, human studies rely on fecal samples for microbiome studies. In addition, feeding RS2, including RPS, to humans leads to an increase in fecal *Ruminoccocaceae* (23, 64, 66–68). This was also observed in rats (50, 62, 69). Interestingly, in one rat study, only HAMS, but not RPS, caused an increase in *Ruminoccocaceae* (50), while in another, HAMS-fed rats had higher levels of *Ruminoccocaceae* in the feces vs. the cecum while *Bacteroides* was equally present in feces and cecum (62), supporting that sampling location as an important variable (61).

*Bifidobacterium* relative abundance was increased in our study as it was in several human studies. In particular, an increase in *Bifidobacterium* (67) was the predominant change observed in one larger study which also used RPS. Mice fed with an AIN-93-based diet with added HAMS showed a large increase in *Bacteroides* and a smaller increase in the *Lachnospiraceae NK4A136 group* (51). Some changes in the microbiome of pigs fed with RS were similar to our findings, including a reduction in the *Rikenellaceae RC9* gut group and increased *Lachnospraceae* (19, 21). A meta-analysis of 24 studies concluded that RS promoted the growth of *Lactobacilli* and *Bifidobacterium* in pigs (20). Both *Lachnospiraceae-* and *Ruminoccocaceae-*affiliated phylotypes increased in the colon of pigs fed with an RS3 (52).

There have been numerous studies examining the effects of consumption of resistant starches, including RS2, on the microbiome and gastrointestinal gene expression in mice fed with a “Western diet” (26, 28, 56, 70, 71). These studies, however, usually employ diets that have high levels of saturated fat in the form of lard with 45–60% of the dietary calories obtained from fat while providing standard levels of other nutrients. These diets diets do not accurately reflect an American diet that characteristically contains a spectrum of fat sources, including mono and polyunsaturated, along with dairy fat in addition to saturated fats found in meats and higher levels of salt. Nevertheless, there were some similarities between our results using the TWD and those of others using the lard-based HFD. We found that consumption of RPS led to decreased α-diversity and a modest increase in *Bifidobacterium* and members of the *Erysipelotrichaceae* family. Others also found a decrease in microbiome diversity upon feeding RS2 alone or in conjunction with an HFD (26, 28, 47, 56). Rats fed with an HFD (45% kcal from fat) and containing 10% RS from RPS had an increase in *Bifidobacterium* compared to rats fed with the HFD (72). The *Erysipelotrichaceae* family was one of the discriminating taxa for the cecal microbiota in mice fed with an HFD (45% kcal from fat) with 20% HAMS (28). Similarly, mice fed with an HFD (60% kcal from fat) with either 5, 15, or 25% RPS or 25% HAMS had an increase in *Bifidobacterium* and *Bacteroides* but had a decrease in *Lachnospiraceae* compared to mice fed with the HFD alone. Overall, increased levels of *Bifidobacterium* and *Bacteroides* appear to be a common microbiota feature when rodents are fed with HAMS.

Multiple factors may account for the differences between our results and those reported by others. Diet composition and length of time on the diets are key variables. As discussed above, our diet differs from other “Western diets” and more accurately reflects the typical American diet. Thus, this is the first study to look at the effect of RS on a rodent diet based on NHANES data (30). Furthermore, mice were fed with the RS diets for only 3 weeks while other rodent studies have used longer times (18, 26, 56, 73), which may result in further changes to the microbiota. Although RS2-type starches share some basic characteristics, there may be subtle differences in their structures that can affect subsequent utilization by various bacterial species (17). Furthermore, how the diet is prepared may also affect the structure of the added RS, including the types of fats, proteins, and other carbohydrates used in the diet preparation. The strain and source of the mice used in the study can also be a factor as it is known that the microbiome of the same strain of mice, along with breeding and housing conditions, differs between vendors (74–76). All these factors may contribute to the differences observed between studies.

Interpreting gene expression studies in a tissue like colon, where there is a mix of parenchymal and non-parenchymal cell types, is challenging. To address this, we first determined whether there were patterns of gene expression associated with specific cell types in the three tissues by three doses of RPS. In the cecum, 66 genes and 47 genes that were upregulated or downregulated, respectively, were associated with individual cell types. In the PC, seven upregulated and three downregulated genes were associated with specific cell types. In the DC, six upregulated and five downregulated genes were associated with specific cell types.

Intestinal stem cells can be induced to differentiate into several cell types in the intestinal epithelium, epithelial cells (ECs), goblet cells, and Paneth cells. These three cell types play a concerted role in maintaining the EC barrier function. In the cecum, we found no goblet cell genes induced by 10% RPS. In contrast, four out of 21 goblet cell restricted genes (Krt7, Retnlb, Tff3, and Agr2) were downregulated by 10% RPS. A different scenario occurred in the PC as no goblet cell genes were upregulated and only one gene, Galnt15, was modestly downregulated by 10% RPS. However, two goblet cell-associated genes, Reg3b and Reg3g, were highly upregulated by 5% and 10% RPS (Table 4). Reg3g is induced by the Th2-associated cytokine IL-33 in ECs (77). Fut2, a fucosyltransferase involved in mucin biosynthesis (78) and associated with Th2-responses (79), was also upregulated (1.7-fold) by 5 and 10% RPS. No goblet cell-associated genes were downregulated by any other dose of RPS in the PC.

**Table 4:** Top 20 differentuially expressed genes in the proximal colon.

**Gene**	**2 vs. 0%**		**5 vs. 0%**		**10 vs. 0%**	
	**FC**	***p*** **(adj)**	**FC**	***p*** **(adj)**	**FC**	***p*** **(adj)**
Igkv2-109					−13.7	5.94E-05
Apoa1					−7.0	1.89E-02
Igkv8-28					−6.2	2.29E-01
Igkv10-94					−6.0	1.39E-02
Angptl7					−4.3	2.32E-02
Hoxa9					−3.6	1.86E-02
Igkv5-48					−3.3	3.51E-02
Slc15a1			−3.0	8.20E-04	−3.2	1.39E-04
Insl5					−3.1	2.58E-02
Rasd2					−2.7	1.57E-02
Rnf152					−2.7	1.94E-02
Ttr					−2.7	3.01E-02
Inmt					−2.6	8.35E-03
Cyp2c68					−2.5	3.02E-02
Cckar			−2.0	1.45E-02	−2.2	8.95E-04
Olfr165					−2.1	6.51E-03
Hoxa7					−2.0	2.51E-02
Gm44220					−2.0	9.08E-03
Tmem86a			−1.9	4.14E-02	−1.9	2.17E-02
Rspo3					−1.8	4.81E-03
Hbegf			2.1	2.65E-02	2.2	4.49E-03
Ido1					2.3	1.24E-03
Lpo			1.9	1.25E-05	2.3	2.59E-10
Gda			2.4	4.25E-04	2.3	3.50E-04
Mogat2					2.3	7.42E-04
Irf7					2.3	1.21E-06
Akr1b7	2.4	1.28E-05	2.4	1.25E-05	2.4	2.27E-06
Plet1os			1.9	2.68E-02	2.4	6.19E-04
Duox2			2.4	1.16E-02	2.4	5.18E-03
Mal			3.5	2.59E-04	2.4	1.21E-02
Prss27			3.2	1.15E-02	2.6	3.30E-02
Wfdc18			2.8	4.43E-03	2.6	5.14E-03
Lypd8l			1.8	4.42E-02	2.9	8.94E-07
Csta2					3.0	2.10E-03
Rdh9			3.5	4.43E-03	3.3	3.42E-03
Gm33424	2.4	4.48E-03	2.8	1.92E-05	3.6	1.49E-09
Duoxa2			3.6	3.87E-02	3.7	1.90E-02
Ceacam12			2.3	5.66E-03	4.1	7.30E-09
Reg3a			4.5	5.66E-03	4.4	3.58E-03
Reg3g			18.6	4.72E-03	11.0	1.90E-02
Reg3b			26.3	2.59E-04	16.7	1.37E-03

An even different situation occurred in the DC, where several genes associated with goblet cells were regulated by RPS. Scnn1g, an epithelial non-voltage-gated sodium channel, was downregulated (-4.2-fold) by 10% PRS in the DC. This gene is repressed by IL-4 in human ECs (80). Muc3a and Clca1, two goblet cell restricted genes, were modestly upregulated (1.7-fold) by 10% RPS in the DC. These genes are induced by Th2-associated cytokines (81, 82). Retnlb, a goblet-cell restricted protein (83), was upregulated almost 90-fold in the DC by 5% RPS. Its expression is increased by Th2 cytokines in other models (84). Slc9a3, a chloride ion transporter (85) essential for mucus production and barrier function in mice (86), was upregulated by 5 and 10% RPS in the DC. Slc9a3 is upregulated by IL-13 in human ECs (87). Ang4 was also induced at a low level in DC by 5% RPS. Some of the goblet cell-associated genes are also expressed by Paneth cells, including Ang4 (88), Reg1a (89), and Reg3b (88).

Resident B and T cells in the follicle-associated epithelium of the cecum and colon, associated with the gut-associated lymphoid tissue (GALT), play an important role in the response to pathogens and food allergens (90). Based on gene expression profiles, we found significant changes in genes associated with B and T cell immunology. Despite this, a full discussion of B cell changes is beyond the focus of this current manuscript. It should be noted that immunoglobulin-associated genes constitute six out of the top 10 upregulated genes in the cecum (Table 3) and eight out of the top 10 genes in the DC (Table 5).

**Table 5:** Top 20 differentially epressed genes in the DC.

**Gene**	**2 vs. 0%**		**5 vs. 0%**		**10 vs. 0%**	
	**FC**	***p*** **(adj)**	**FC**	***p*** **(adj)**	**FC**	***p*** **(adj)**
Cyp11a1					−12.8	7.46E-04
Gm17322					−7.3	6.42E-03
Scnn1g					−4.2	1.73E-02
Gm44756/Klk15					−3.1	4.86E-03
Klk15					−3.1	1.33E-04
Scnn1b					−2.9	3.13E-03
Hkdc1					−2.9	3.36E-02
Mt1					−2.8	1.48E-02
Tchh					−2.6	8.48E-03
Ces2b					−2.6	2.94E-02
Lipk					−2.5	2.88E-02
Cyp2w1					−2.5	4.86E-03
Sptbn2					−2.4	1.51E-03
Fn3k					−2.3	2.71E-02
Cyp2u1					−2.3	2.91E-02
Sowaha					−2.3	9.08E-03
D630011A20Rik					−2.3	7.88E-03
Insrr					−2.2	2.91E-02
Brme1					−2.2	1.73E-02
AI182371					−2.1	2.35E-02
Zbp1			3.5	4.06E-02	3.4	1.68E-02
Gm5431					3.5	1.25E-03
Gm20754			2.6	3.88E-02	3.7	1.03E-04
Gm42870					3.9	2.19E-02
Iglc3					4.0	3.02E-02
Dhrs9					4.2	4.06E-02
Mgat4c					4.3	3.13E-03
Gm47914					4.3	2.75E-02
Iglc1					4.4	3.18E-02
Pla2g2a					5.3	3.11E-02
Ighv1-42					6.7	3.03E-02
Ighv1-81					7.6	4.86E-03
Ighg1					8.2	1.96E-03
Igkv4-57	14.1	2.35E-02			8.9	8.01E-03
Gm47465					9.7	1.34E-02
Igkv1-88					9.7	3.19E-02
Ighv4-1					10.4	1.03E-02
Igkv6-25					10.7	7.50E-04
Ighv9-3					10.9	1.18E-02
Slc9a3			9.4	1.07E-02	11.1	8.91E-04
Ighv1-77					21.7	1.91E-02

T cells can be polarized to several phenotypes *in vitro* and *in vivo*. Cytokines, like interferon-gamma (IFN-g, a type 2 IFN), leads to Th1 polarization. Th1-polarized T cells are involved in the immune response during inflammation. Th2 T cells are at the other end of the polarization spectrum and can be induced *in vitro* by cytokines like IL-4 and IL-13. There is evidence for a Th1-type response based on the large number of genes that are regulated by type 1 or type 2 IFNs. The balance of genes that were differentially regulated by RPS in the cecum favors a Th1-like response. Four (Adcy8, Ly6g6c, Plscr1, Hif1a) and 18 genes (Psmb9, Ifit2, Parp9, Trim34a, Uba7, Irf8, Clec2h, Oas1h, Samd4, Ifit3b, Mx1, Dtx3l, Ifit1bl2, Oas3, Mal, Irf7, Mov10, Ifit1b) regulated by type 1 IFNs were downregulated and upregulated, respectively, by 10% RPS. In addition, five (Psmb9, Parp9, Siglec1, Psmb8, Gvin1) type 2 IFN-induced genes were upregulated by 10% RPS. Other genes associated with a Th1 response (Klrd1, Pim1) were also upregulated by 10% RPS. This trend is less evident for 5% RPS as three and five genes regulated by type 1 IFNs were downregulated and upregulated, respectively. In the PC, Angptl7 and 2 upregulated genes (Irf7, Mal) by type 1 IFNs were downregulated and upregulated, respectively, by 10% RPS. One type 2-induced (Ido1) and two Th1-associated genes (Ceacam1, Bcl3) were upregulated by 10% RPS. In the DC, seven (Irf1, Rsad2, Cgas, Ifit1bl1, Isg15, Mx1, Oas3) type 1 IFN-induced genes were downregulated and upregulated, respectively, by 10% RPS. Three (Cxcl10, Psmb8, Irf1) type 2-induced and two Th1 associated (Duox2, Bcl3) genes were upregulated by 10% RPS.

There is more consistent evidence of Th2 cell-polarized response based upon a fairly large number of genes that are regulated by RPS and are also regulated by Th2 cytokines, particularly in the PC and DC. Some of these genes and their function are discussed above. In the cecum, only two (Itln1, Muc3a) out of 476 upregulated genes were associated with a Th2 response. In the PC, five (Fut2, Ikzf2, Reg3g, Sprr2a2, St6gal1) out of 76 genes (7%) that were upregulated by 10% RPS were Th2-associated. In the DC, three (Clca1, Muc3a, Slc9a3) out of 136 genes were regulated by 10% RPS. Socs3, a Th2-associated gene (91) and negative regulator of multiple JAK-STAT signaling pathways (92), was highly upregulated by 5% RPS in the DC. The magnitude of fold-change (38.2) was the second highest regulated by any dose of RPS in all three tissues (Supplementary Table 10). No genes associated with a Th2-response were downregulated in any tissue by any dose of RPS except for the small downregulation of Retnlb by 10% RPS in the cecum. Even though butyrate has been shown to induce differentiation of colonic Treg cells (93), there was very limited evidence of regulation of any gene associated with a Treg or Th17 response.

Macrophages (Mϕs) represent around 5–15% of the non-parenchymal cell population in almost every tissue. Similar to T cells, Mϕs can be polarized to a pro-inflammatory (M1) or anti-inflammatory (M2) phenotypes *in vitro* by the use of cytokines and microbial or parasitic products. However, the extremes of polarization observed in cell culture are rarely observed *in vivo* (94). In our model where butyrate levels were elevated by increasing dietary RPS (in the cecum and likely in the colon), there was limited evidence that polarized Mϕs contributed to the change in gene expression. Thus, cell type analysis revealed that many changes in gene expression in the three tissues by 5 and 10% RPS could be associated with specific cell types, namely, goblet cells, B cells, and T cells. Regarding T cells, there was evidence for both Th1 and Th2 responses depending on the level of dietary RPS or tissue.

The first line of defense in the colon is the ECs that can sense microbial products and secrete anti-microbial peptides/proteins, including Ang4, Retnlb, Reg3b, and Reg3g (95). Dysbiosis-induced chronic activation of ECs can exacerbate colon inflammation (96). Three additional genes, involved in antibacterial responses, were upregulated in all three tissues (Tables 6–8) by 10% RPS vs. control (Casp4, Duox2, and Duoxa2). Other than these changes, the three tissues had three different gene sets associated with antibacterial responses to RPS. In the cecum, several genes involved in oxygen or nitric oxide-dependent intracellular killing, Duoxa1 (3.9-fold), Duoxa2 (3.9-fold), Duox2 (6.7-fold), and Nos2 (6.5-fold), were the genes that were most upregulated in the 10% RPS group. In the PC, six genes involved in antibacterial responses were upregulated by 5% RPS [Duox2 (2.4-fold), Lpo (1.9-fold), Plac8 (1.5-fold), Reg3a (4.5-fold), Reg3b (26.3-fold), Reg3g (18.6-fold)]. All of these, except Plac8, were upregulated to a lesser degree by 10% RPS. Reg3b has antibacterial activity against Gram-negative Salmonella enteritidis but not Gram-positive *Listeria monocytogenes* (97). In contrast, Reg3g has antibacterial activity against gram-positive bacteria but not Gram-negative *Escherichia coli* (98).

**Table 6:** Selected differential expressed genes in the cecum.

**Response**	**Gene**	**5 vs. 0%**		**10 vs. 0%**	
		**FC**	***p*** **(adj)**	**FC**	***p*** **(adj)**
Antibacterial /antiparasitic	Areg			−2.5	1.17E-02
	Defb37	−2.3	8.73E-02	−2.4	2.94E-02
	Il11			−1.9	3.68E-02
	Retnlb			−1.7	4.07E-02
	Tff3	−1.4	1.04E-02	−1.6	2.38E-05
	Nos3			−1.6	2.72E-02
	Bdkrb2			−1.5	1.15E-02
	Irgm2			1.5	7.59E-03
	Noxa1	1.4	1.58E-04	1.6	7.87E-11
	Tlr2			1.6	4.18E-03
	Atp7a			1.6	2.91E-02
	Casp4	1.7	1.30E-03	2.1	6.33E-08
	Retnla	2.7	2.20E-07	3.3	6.55E-12
	Duoxa1			3.9	1.09E-02
	Duox2	2.7	5.95E-02	3.9	5.69E-04
	Nos2	2.9	3.67E-02	6.5	4.23E-07
	Duoxa2	4.4	1.14E-02	6.7	8.27E-05
Antiviral	Ly6g6c	−1.9	9.73E-03	−2.4	1.78E-05
	Adcy8			−1.7	3.68E-02
	Plscr1	−1.4	5.56E-02	−1.7	6.93E-05
	Hif1a	−1.5	2.58E-02	−1.5	7.59E-03
	Fkbp5			−1.5	3.21E-02
	Wfdc1			1.5	2.19E-02
	Psmb9			1.5	1.56E-02
	Ifit2			1.5	4.50E-02
	Parp9			1.5	4.97E-04
	Trim34a			1.6	3.79E-02
	Uba7			1.6	2.62E-03
	Irf8			1.6	1.84E-02
	Clec2h	1.4	5.15E-02	1.6	6.78E-05
	Oas1h	1.7	1.71E-02	1.7	5.52E-03
	Samd4			1.8	5.96E-03
	Ifit3b			1.8	4.51E-02
	Siglec1			1.8	5.34E-03
	Mx1			1.8	2.89E-05
	Psmb8			1.8	2.85E-02
	Dtx3l			1.9	1.28E-04
	Ifit1bl2			1.9	1.25E-03
	Oas3			2.0	4.80E-03
	Mal	1.6	9.29E-03	2.0	1.97E-06
	Zbp1			2.1	1.62E-02
	Gvin1			2.3	2.91E-02
	Irf7	1.6	2.75E-02	2.3	5.27E-07
	Mov10	1.9	1.72E-04	2.8	3.83E-12
	Ifit1b			3.5	1.25E-02

**Table 7:** Selected differential expressed genes in the proximal colon.

**Response**	**Gene**	**5 vs. 0%**		**10 vs. 0%**	
		**FC**	***p*** **(adj)**	**FC**	***p*** **(adj)**
Antibacterial/antiparasitic	Lpo	1.9	1.25E-05	2.3	2.59E-10
	Duox2	2.4	1.16E-02	2.4	5.18E-03
	Reg3a	4.5	5.66E-03	4.4	3.58E-03
	Reg3g	18.6	4.72E-03	11.0	1.90E-02
	Reg3b	26.3	2.59E-04	16.7	1.37E-03
	Plac8	1.5	1.48E-02		
	Retnlb	1.8	3.18E-02	1.7	1.90E-02
Antiviral	Angptl7			−4.3	2.32E-02
	Ifit1			1.6	4.27E-02
	Zbp1	2.1	2.01E-02	2.1	1.38E-02
	Ido1			2.3	1.24E-03
	Irf7			2.3	1.21E-06
	Mal	3.5	2.59E-04	2.4	1.21E-02
	Trim15	2.6	1.35E-02		

**Table 8:** Selected differential expressed genes in the distal colon.

**Response**	**Gene**	**5 vs. 0%**		**10 vs. 0%**	
		**FC**	**p (adj)**	**FC**	**p (adj)**
Antibacterial/antiparasitic	Duox2			1.9	9.08E-03
	Cxcl10			2.5	1.18E-02
	Pla2g2a			5.3	3.11E-02
	Ang4	1.6	2.44E-02		
	Retnlb	87.8	2.48E-03		
Antiviral response	Sun2			−1.6	3.64E-02
	Nlrp6			1.5	4.50E-02
	Irf1			1.6	4.26E-06
	Rsad2			1.7	3.07E-02
	Oasl2			1.7	3.52E-02
	Cgas			1.7	2.96E-02
	Ifit1bl1			2.0	4.13E-04
	Isg15			2.0	2.24E-02
	Mx1			2.3	3.73E-03
	Oas3			3.2	3.87E-03
	Zbp1	3.5	4.06E-02	3.4	1.68E-02
	Gm5431			3.5	1.25E-03
	Trim15	4.0	2.44E-02		
	Mfsd2a	4.0	2.44E-02		

Genes like Ang4, Reg3a, Reg3b, Reg3g, and Retnlb also play a role in antiparasitic responses (99, 100). In the cecum, three additional genes associated with antiparasitic responses (Areg, Il11, Tff3) were downregulated by 10% RPS, and only one gene was upregulated by 10% RPS. However, in PC, Retnlb was modestly upregulated by 5% (1.8-fold) and 10% RPS (1.7-fold). In contrast, in the DC, Retnlb was highly upregulated (87.8-fold) by 5% RPS. In fact, it was the gene that exhibited the highest level of regulation by RPS in all three datasets. In the DC, only one additional gene, Ang4, involved in antiparasitic responses was upregulated at a low level.

In addition to antibacterial and antiparasitic responses, RPS influenced many genes associated with antiviral responses in the three tissues. The balance of genes that were differentially regulated by RPS in the cecum favors an antiviral response as two (Adcy8, Plscr1) and nine (Fkbp5, Wfdc1, Uba7, Irf8, Oas1h, Mx1, Dtx3l, Oas3, and Irf7) genes with demonstrated antiviral activity were downregulated and upregulated, respectively, by 10% RPS. In the PC, four genes (Ifit1, Zbp1, Irf7, and Trim15) with demonstrated antiviral activities were upregulated by 10% RPS. In the DC, one (Sun2) and nine (Irf1, Rsad2, Oasl2, Cgas, Isg15, Mx1, Oas3, Zbp1, and Trim15) genes with demonstrated antiviral activity were downregulated and upregulated, respectively, by 10% RPS.

Other functionally associated classes of genes that are overrepresented in upregulated genes in the 10% RPS group include extracellular matrix/structural proteins. Multiple genes involved in carbohydrate, lipid, mineral, and vitamin metabolism were also regulated by RPS in all three tissues. A full discussion of these genes is beyond the scope of the current manuscript.

Several vitamins are important regulators of mucosal immunity. Chief among these are vitamin A (VA) and vitamin D (VD) which can influence the differentiation of gut epithelial cells, T cells, B cells, and macrophages (101). In the cecum, five genes [Dhrs9 (-2.4-fold), Rbp1 (-1.6-fold), Rbp2 (-2.8-fold), and Rbp7 (-2.8-fold)] involved in VA transport/metabolism were downregulated by 10% RPS group. In the PC, 2 genes, Rdh16 (2.0-fold), Rdh9 (3.3-fold), and one gene, Adh1 (-1.6), were up and downregulated, respectively, by 10% RPS. Similarly, in the DC, 2 genes Rdh9 (DC, 3.3), Dhrs9 (DC, 4.2), Cyp2w1 (DC,−2.5) were up and downregulated respectively, by 10% RPS. It is difficult to argue for increased or decreased VA activity based on gene expression in our model because the expression pattern of VA-regulated genes appears to be unrelated to these gene expressions of RPS-influenced genes.

Cyp24a1 was the most downregulated gene in any tissue (-25.8) at any level of RPS (Table 5). Cyp24a1 is induced by vitamin D (VD) and is involved in its catabolism (102). Like the situation with VA described above, it is difficult to argue for increased or decreased VD activity based on gene expressions in our model because aside from Cyp24a1, there were no other VD-induced genes. In addition, increased levels of one VD-induced gene (Alox5) were found in the cecum of animals fed with 10% RPS. Three VD-induced genes (Atp2b1, Cldn2, and Tmem37) were upregulated, and one VD-induced gene (Alox5) was downregulated by 10% RPS in the PC. Similarly, three VD-induced genes (Alox5, Cxcl10, and Cd274) were upregulated, and one (Cyp27a1) VD-induced gene was downregulated by 10% RPS in the DC.

These data indicate that RPS has wide-ranging effects on genes involved in immunity and metabolism. The gene expression patterns that we discovered indicate that RPS may prime the immune response to bacteria, helminth parasites, and viruses. To our knowledge, the patterns we found of gene expression in our study are unique. Two other studies had previously shown that type 2 RS results in an increase of a very small number of genes that were regulated by RPS in our study. Rats fed with a 30% HAMS (on a purified chow background diet) only exhibited an increase in four genes (Areg, Asns, Casp4, and Hif1a; microarray/RT-PCR) that overlap with genes induced by RPS in our study (103). Similarly, mice fed with a diet containing 36% HAMS (total type 2 RS 20%) on an HFD background had higher expression of Tlr2 and Nod2 (determined by real-time PCR) in the cecum compared with animals fed with the HFD alone (28). A small number of studies suggest that manipulation of the microbiome by feeding microorganisms or foodstuffs yield similar results to ones described in the current manuscript. In one study, feeding *Lactobacillus delbrueckii* or yogurt to mice led to increased Reg3g expression in the small intestine (104).

The PCA of the gene expression data showed a clear separation of the four dietary groups in the cecum. These dietary groups correlated with a large number of differentially expressed genes in the cecum that was most prevalent in the 10% RPS group. Both the number of differentially expressed genes and the group separation in PCA plots decreased in the PC and DC along with the number of differentially expressed genes. In mice, the cecum is large (105) and is the primary site of fermentation of fibers and RS. Fermentation progressively decreases from the proximal to the distal colon. The tissue-dependent decrease in differentially expressed genes parallels the decrease in fermentation. This is in contrast to humans where the cecum is small (106) and fermentation primarily occurs in the PC. We also noted that most of the differentially expressed genes in tissue were unique and were not shared between tissues, suggesting that RPS induces location-specific changes in gene expression.

There are several limitations to our studies. First, there was no comparison made to the AIN-93 diet. Second, both diets, TWD and AIN-93, and the AIN-76 diets also contain casein as the protein source and cellulose as the fiber source. A second TWD has been developed using whole food ingredients. This reflects typical American intake profiles for major protein, carbohydrate, and fiber sources by employing NHANES dietary surveys (as for the original TWD) in conjunction with commodity intake data. This refinement accounts for varied fiber contents and other bioactives present in whole foods that may contribute to health and disease (31). We intend to use a modified version of this diet in future studies. We recognize that the findings described in the manuscript are derived from a single time course. We are currently conducting shorter- and longer-term studies.

## Conclusion

The results presented here demonstrate that feeding mice with RPS using a basal diet that emulates a typical American diet can have significant effects on both the microbiome and gene expression in the cecum. We found that feeding RPS promoted growth of some but not all genera associated with SCFA production from resistant starches. Factors affecting the selection of certain bacterial genera over others also capable of fermenting resistant starches may be due in part to the basal microbiota composition, but other factors are likely to come into play and further research will be required to elucidate these mechanisms. In addition, we found that RPS feeding significantly impacted host gene expression in the cecum, PC, and DC. The number of differentially expressed genes was highest in the cecum and decreased in the PC and DC where fermentation is reduced. Genes associated with anti-bacterial, anti-viral, and anti-parasite immune responses were upregulated in all three tissues, indicating that RPS be priming the immune system for resistance to infection.

## Data Availability Statement

The datasets presented in this study can be found in online repositories. The names of the repository/repositories and accession number(s) can be found in the article/Supplementary Material.

## Ethics Statement

The animal study was reviewed and approved by Institutional Animal Care and Use Committee, USDA/ARS Beltsville Agricultural Research Center.

## Author Contributions

AS and HD conceived and designed this experiment and supervised all experimental works. AS, CC, LC, RW, and HD performed experiments. AS, CC, RW, and HD performed data analysis. AS, CC, and HD drafted the manuscript. AS, CC, RW, KH, and HD helped to revise the manuscript. All authors have read and approved the final manuscript.

## Funding

This work was supported by the USDA ARS Project 8040-53000-021-00D.

## Conflict of Interest

The authors declare that the research was conducted in the absence of any commercial or financial relationships that could be construed as a potential conflict of interest.

## Publisher's Note

All claims expressed in this article are solely those of the authors and do not necessarily represent those of their affiliated organizations, or those of the publisher, the editors and the reviewers. Any product that may be evaluated in this article, or claim that may be made by its manufacturer, is not guaranteed or endorsed by the publisher.

## Abbreviations

DC: Distal colon
EC: Epithelial cell
HFD: High fat diet
HAMS: high-amylose maize starch
NHANES: National Health and Nutrition Examination Survey
Mϕ: Macrophage
PC: Proximal colon
RS: Resistant starch
RPS: Resistant potato starch
Treg: Regulatory T cell
TWD: Total Western diet
VA: Vitamin A
VD: Vitamin D.
