# Supplementary material for: Resistant Potato Starch Alters the Cecal Microbiome and Gene Expression in Mice Fed a Western Diet Based on NHANES Data
Source: Front Nutr. 2022 Mar 22;9:782667. doi: 10.3389/fnut.2022.782667 (PMC8983116; doi:10.3389/fnut.2022.782667)
Supplement: Supplementary file 11 [file Data_Sheet_1.PDF]

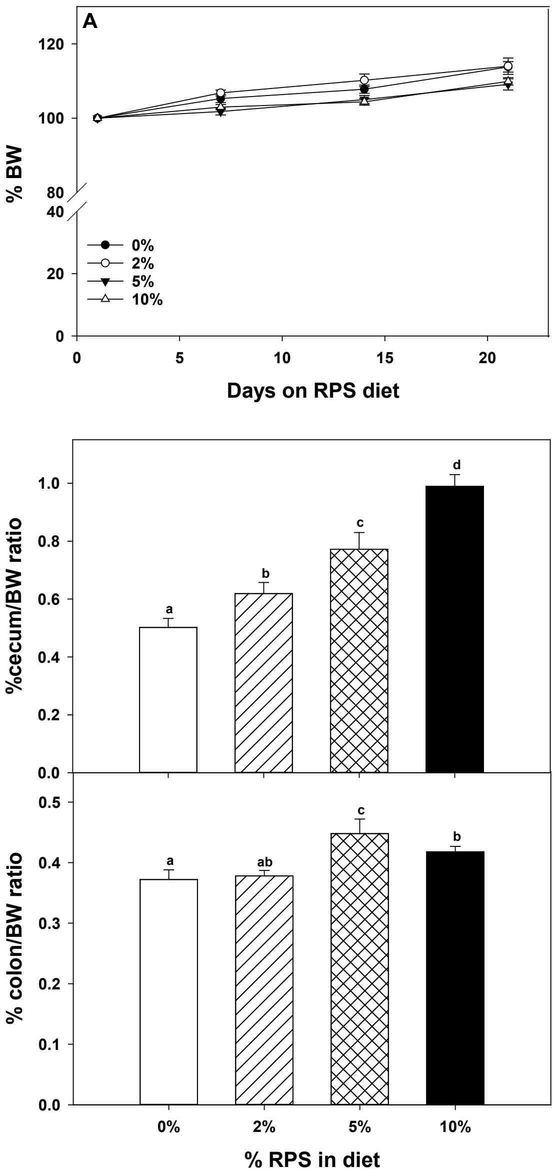

Figure 1S. Feeding mice RPS increases cecum and colon/BW ratios.

Mice were fed the basal TWD for 6 weeks. Subsets of mice continued to receive the TWD or were fed the TWD plus 2, 5, or 10% RPS for an additional three weeks. Panel A. Mice were weighed weekly after switching to the RPS containing diets and body weights (BW) are expressed as the %BW normalized to the BW on the day the mice were switched to the RPS diets (100%, Panel A) and are expressed as the Mean±SEM, n=9-10. The cecum was removed and weighed and expressed as the % cecum/BW. The terminal 6 cm of colon was excised, fecal pellets removed and weighed. Data is from two replicate experiments and are expressed as Mean ± SEM of the % tissue to body weight ratio (1A-cecum, 1B colon), n=16-17mice/group. Groups with different letters are significantly different, $p < 0.05$, by ANOVA (Holm-Sidak).
