# Supplementary material for: Resistant Potato Starch Alters the Cecal Microbiome and Gene Expression in Mice Fed a Western Diet Based on NHANES Data
Source: Front Nutr. 2022 Mar 22;9:782667. doi: 10.3389/fnut.2022.782667 (PMC8983116; doi:10.3389/fnut.2022.782667)
Supplement: Supplementary file 12 [file Data_Sheet_2.PDF]

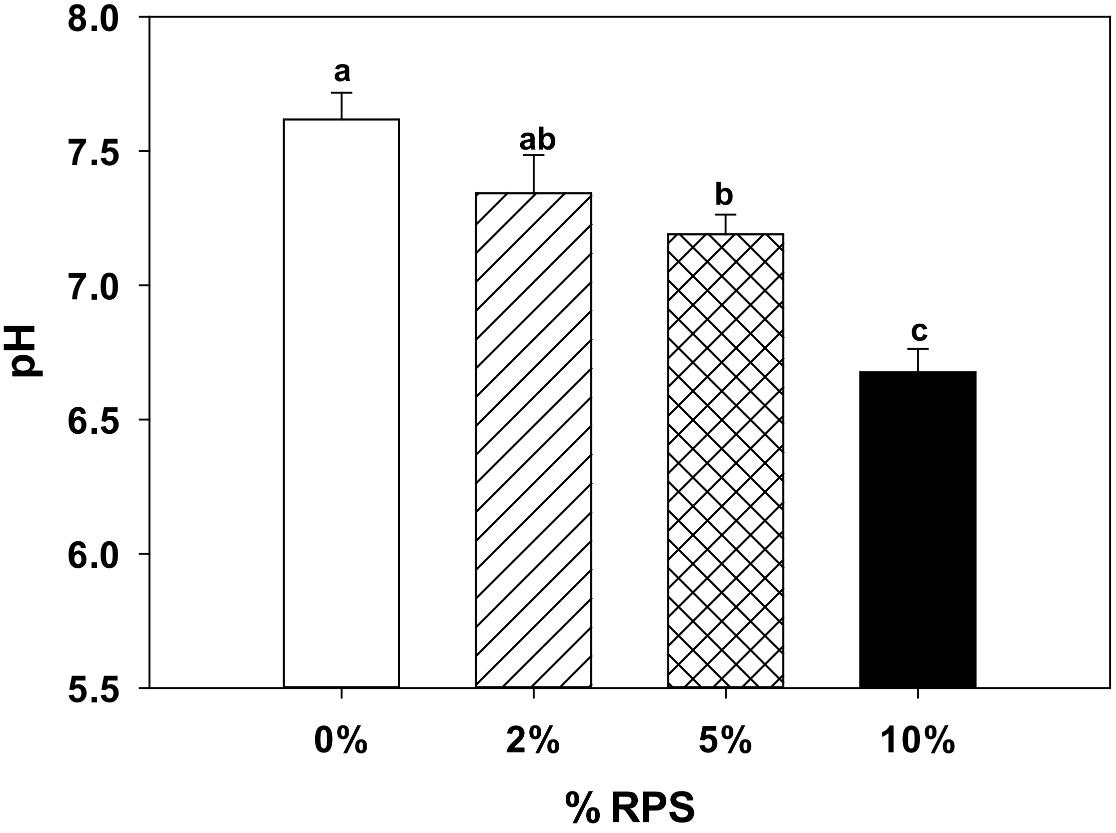

Figure 2S. Feeding mice RPS decreases fecal pH.

Fecal pellets were collected from mice fed the TWD or TWD plus 2, 5, or 10% RPS. Pellets were homogenized in 5 (w/v) volumes water, the solid material removed by centrifugation and the pH of the resulting supernatant determined. Data is expressed as Mean ± SEM, n=9-10 mice/group. Groups with different letters are significantly different, $p < 0.05$, by ANOVA (Holm-Sidak).
