# Supplementary material for: Resistant Potato Starch Alters the Cecal Microbiome and Gene Expression in Mice Fed a Western Diet Based on NHANES Data
Source: Front Nutr. 2022 Mar 22;9:782667. doi: 10.3389/fnut.2022.782667 (PMC8983116; doi:10.3389/fnut.2022.782667)
Supplement: Supplementary file 13 [file Data_Sheet_3.PDF]

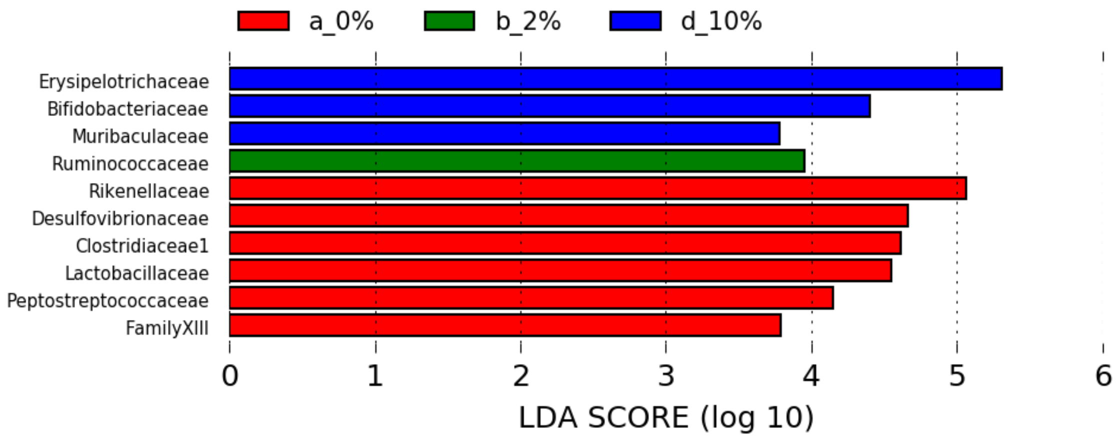

Figure 3S. LEfSe analysis identifies differentially abundant families as potential biomarkers. 16S sequencing data from the cecal microbiome of mice fed the TWD or TWD plus 2, 5 or 10% RPS were analyzed by the LEfSe method using Kruskal-Wallis test ($P < 0.05$) with LDA score > 2.0 being considered significant. N=8-9 mice/group.
