# Supplementary material for: Resistant Potato Starch Alters the Cecal Microbiome and Gene Expression in Mice Fed a Western Diet Based on NHANES Data
Source: Front Nutr. 2022 Mar 22;9:782667. doi: 10.3389/fnut.2022.782667 (PMC8983116; doi:10.3389/fnut.2022.782667)
Supplement: Supplementary file 14 [file Data_Sheet_4.pdf]

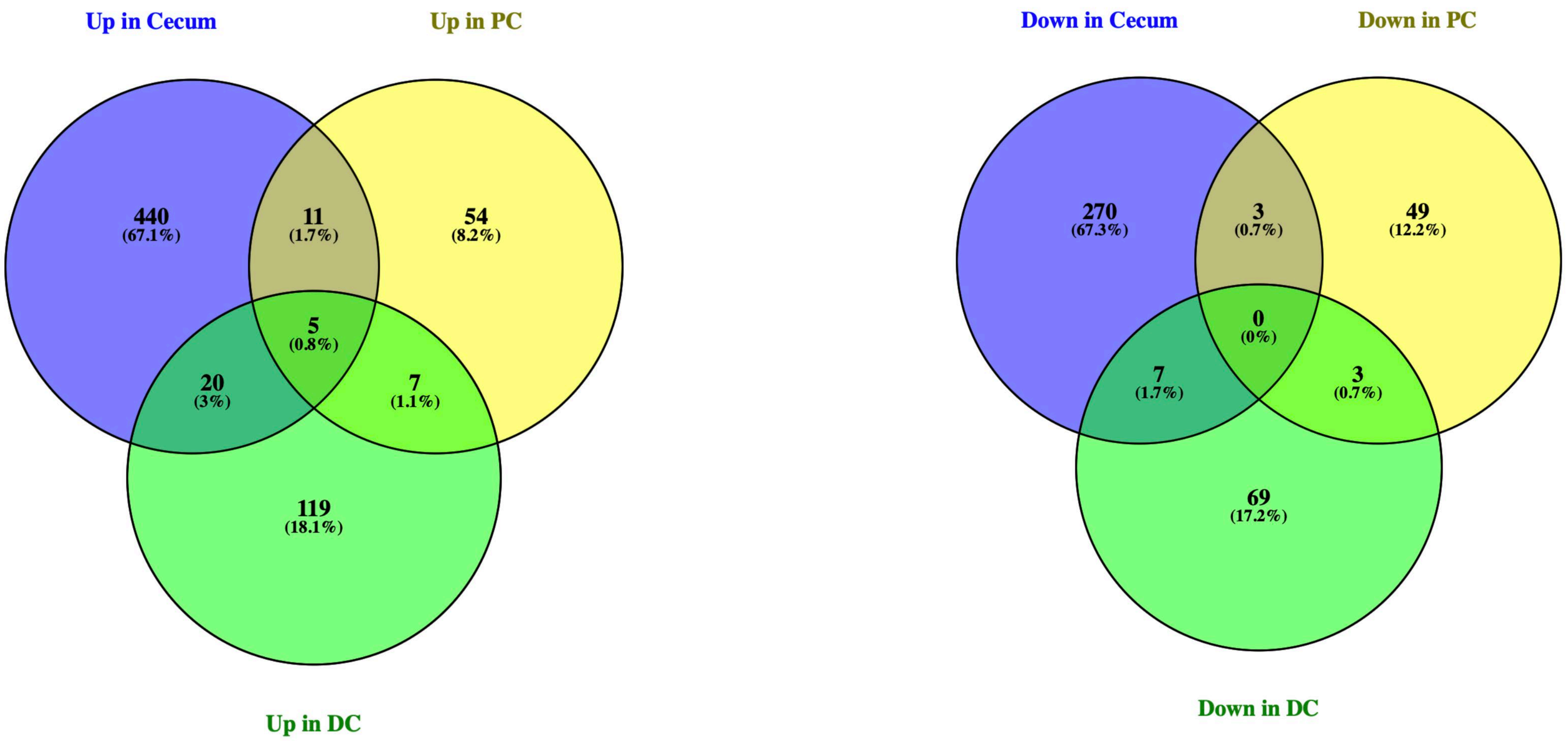

Figure 4S. Venn Analysis of Genes Commonly Upregulated (A) and Downregulated (B) by 10% RPS in Cecum, Proximal Colon (PC) and Distal Colon (DC). Differentially expressed genes (up- or downregulated >1.5 fold at a FDR adjusted p value < 0.05) in each tissue in animals fed 10% RPS, were analyzed by Venn analysis using the online tool Venny 2.1 (https://bioinfogp.cnb.csic.es/tools/venny/).
